# Supplementary material for: Synthesis and Antimicrobial Evaluation of Some New Organic Tellurium Compounds Based on Pyrazole Derivatives
Source: Molecules. 2020 Jul 29;25(15):3439. doi: 10.3390/molecules25153439 (PMC7435781; doi:10.3390/molecules25153439)
Supplement: Supplementary file 1 [file molecules-25-03439-s001.pdf]

molecules

MDPI

Synthesis and Antimicrobial Evaluation of Some New Organic Tellurium Compounds Based on Pyrazole Derivatives

Asmaa B. Sabti [1], Adil A. Al-Fregi [2,*] and Majeed Y. Yousif [2]

[1] Department of Chemistry, College of Science, University of Misan, Basrah 62001, Iraq; asmaabadr86.ab@gmail.com

[2] Department of Chemistry, College of Science, University of Basrah, Basrah 61004, Iraq; majeedyousef@gmail.com

* Correspondence: dr.adilalfregi@gmail.com or dr.adilalfregi@uobasrah.edu.iq

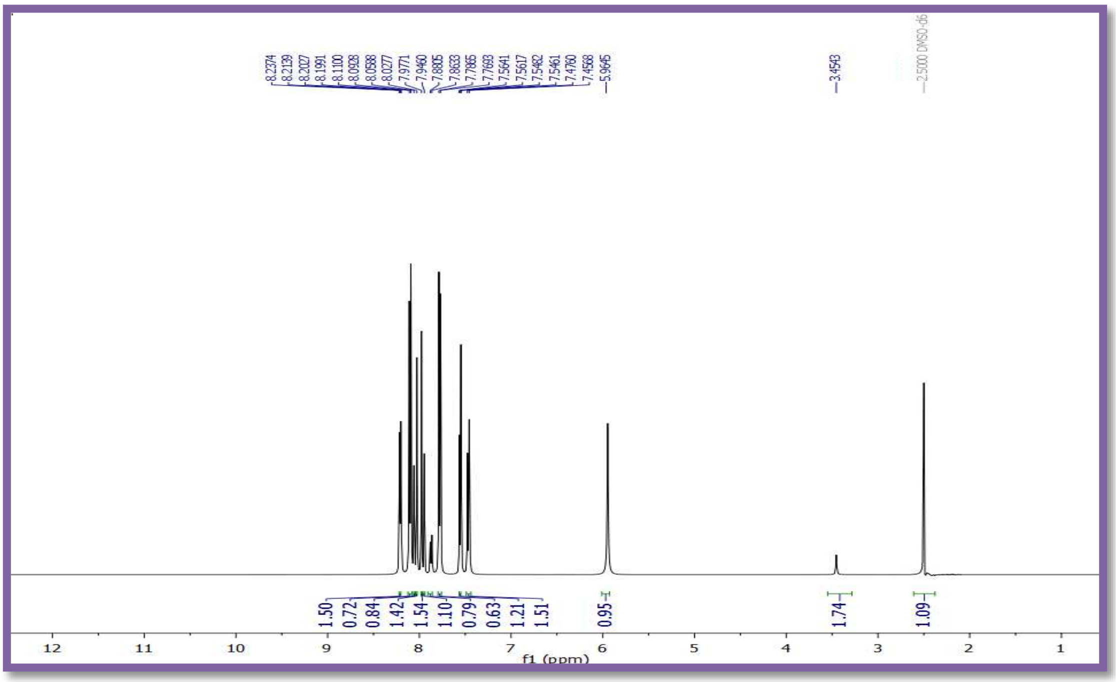

Figure S1. ^{1}H NMR spectrum of 2-(3-(4-Bromophenyl)-5-(2-chlorophenyl)-1H-pyrazol-1-yl)-3,5-dinitrophenyl) mercury (II) chloride (1).

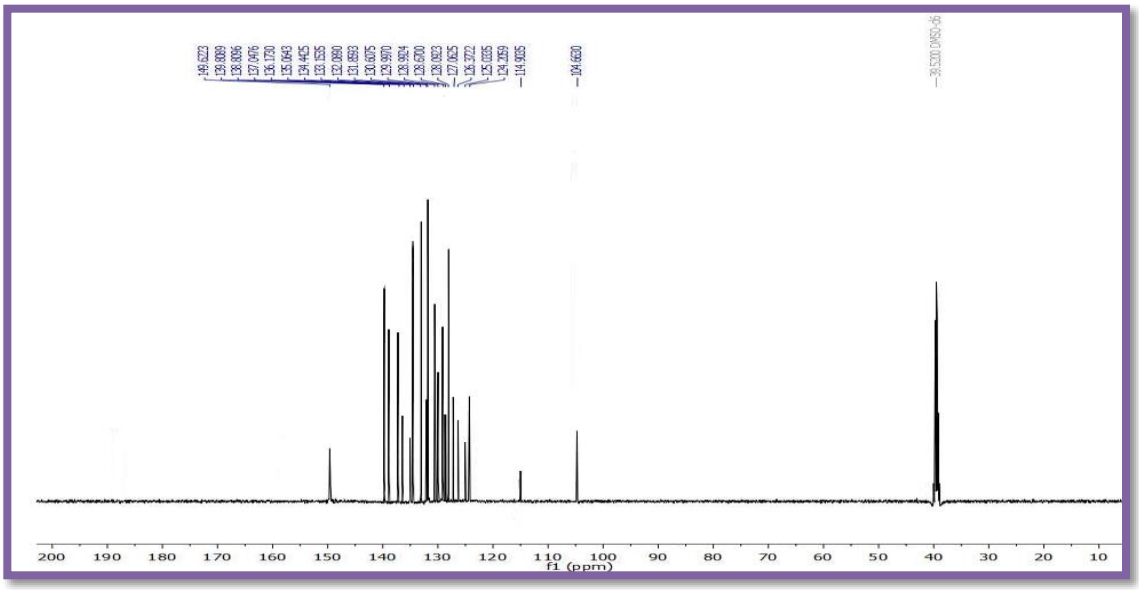

Figure S2. ^{13}C NMR spectrum of 2-(3-(4-Bromophenyl)-5-(2-chlorophenyl)-1H-pyrazol-1-yl)-3,5-dinitrophenyl) mercury (II) chloride (1).

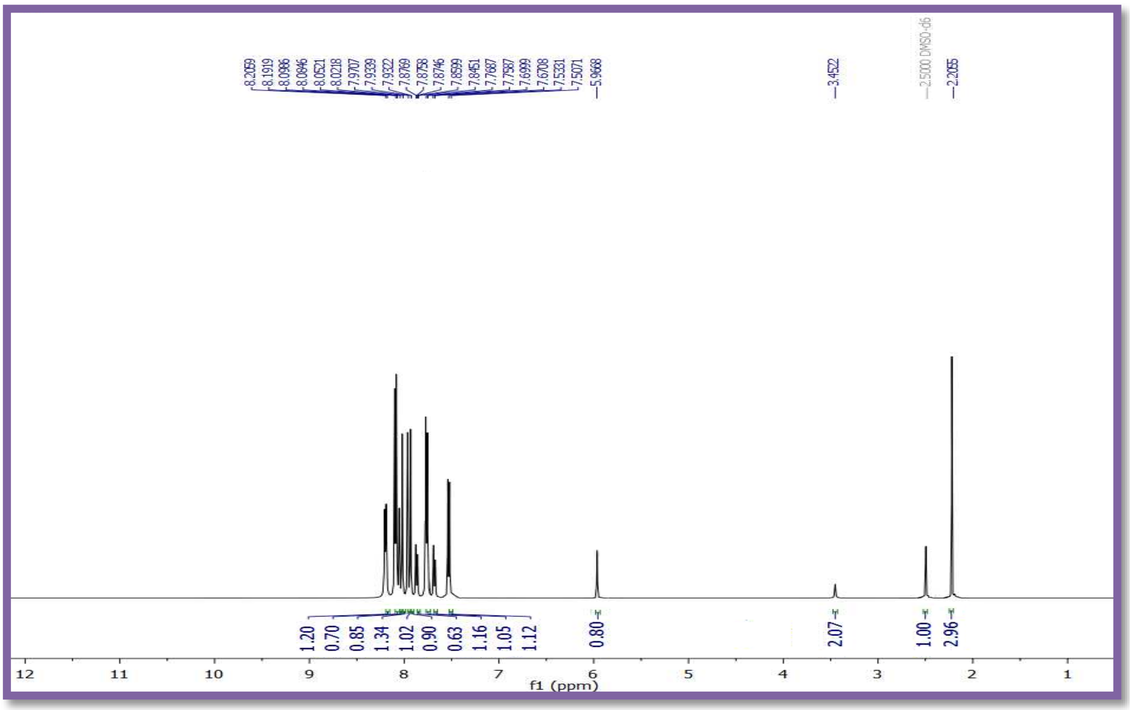

Figure S3. ^{1}H NMR spectrum of 2-(5-(2-chlorophenyl)-3-(4-methyl phenyl)-1H-pyrazol-1-yl)-3,5-dinitro phenyl) mercury(II) chloride (2).

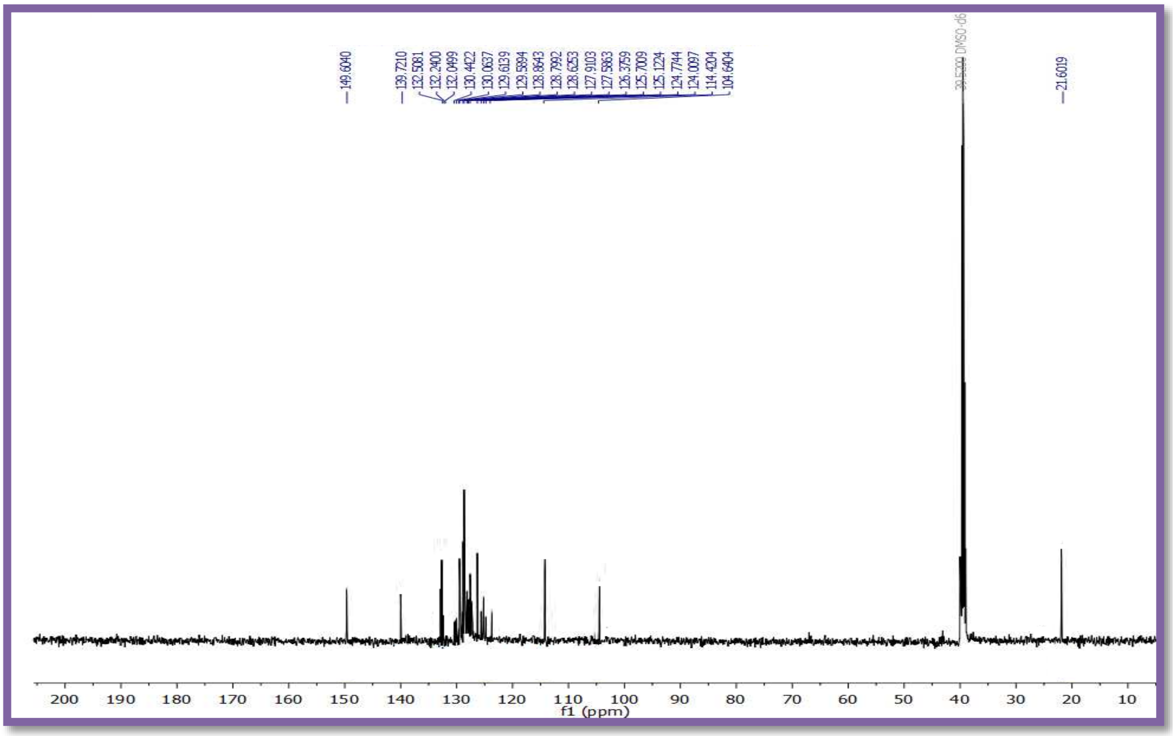

Figure S4. ^{13}C NMR spectrum of 2-(5-(2-chlorophenyl)-3-(4-methyl phenyl)-1H-pyrazol-1-yl)-3,5-dinitro phenyl) mercury(II) chloride (2).

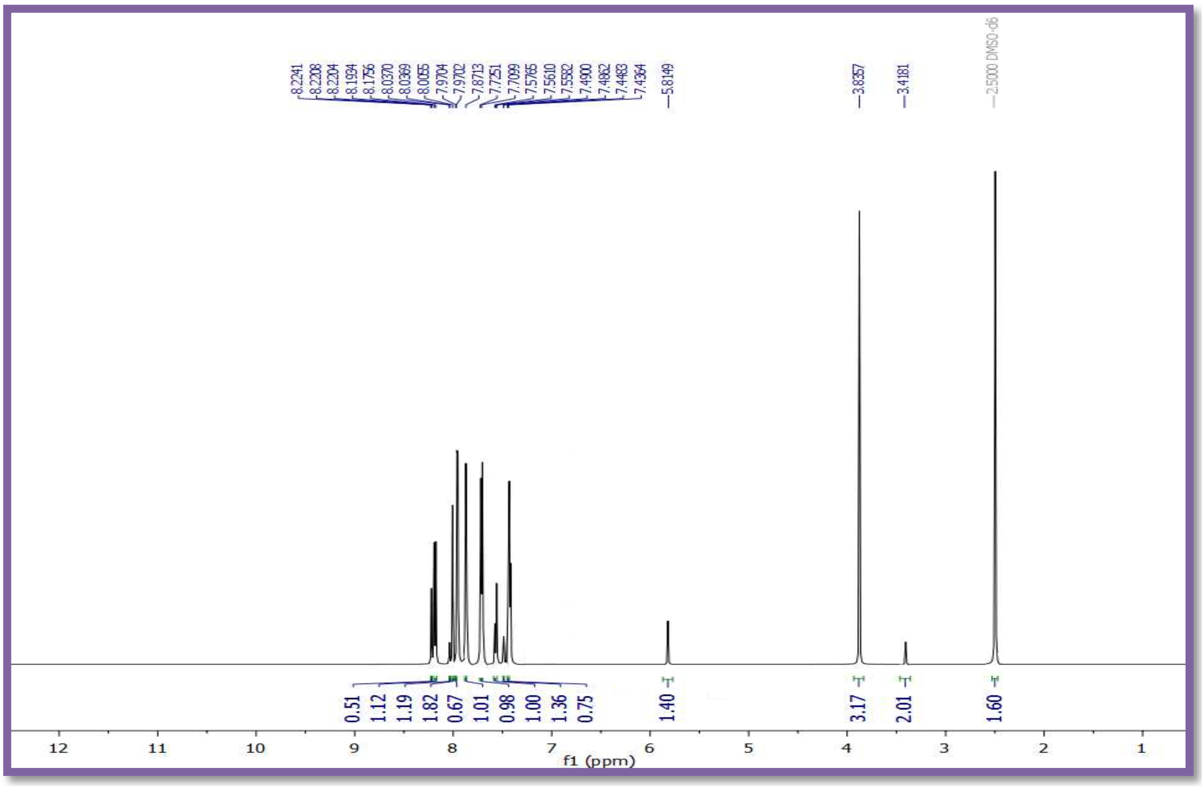

Figure S5. ^{1}H NMR spectrum of 2-(5-(2-chlorophenyl)-3-(4-methoxyphenyl)-1H-pyrazol-1-yl)-3,5-dinitrophenyl)mercury(II) chloride(3).

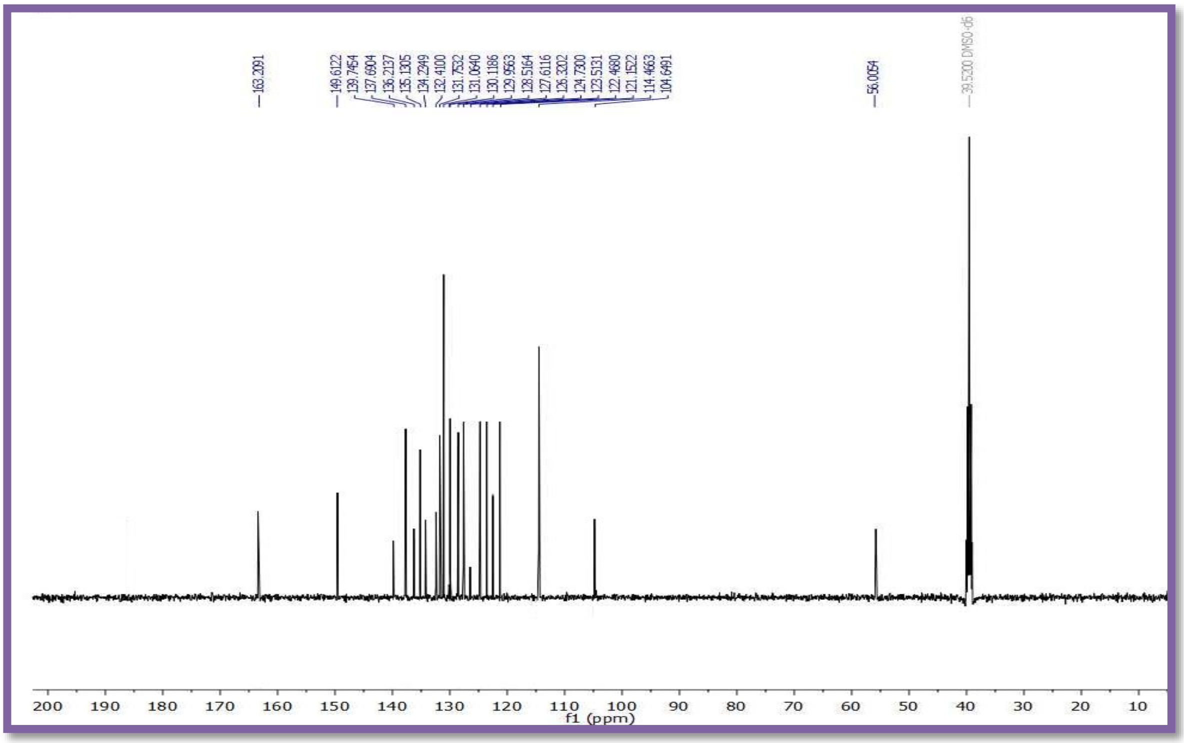

Figure S6. ^{13}C NMR spectrum of 2-(5-(2-chlorophenyl)-3-(4-methoxyphenyl)-1H-pyrazol-1-yl)-3,5-dinitrophenyl)mercury(II) chloride(3).

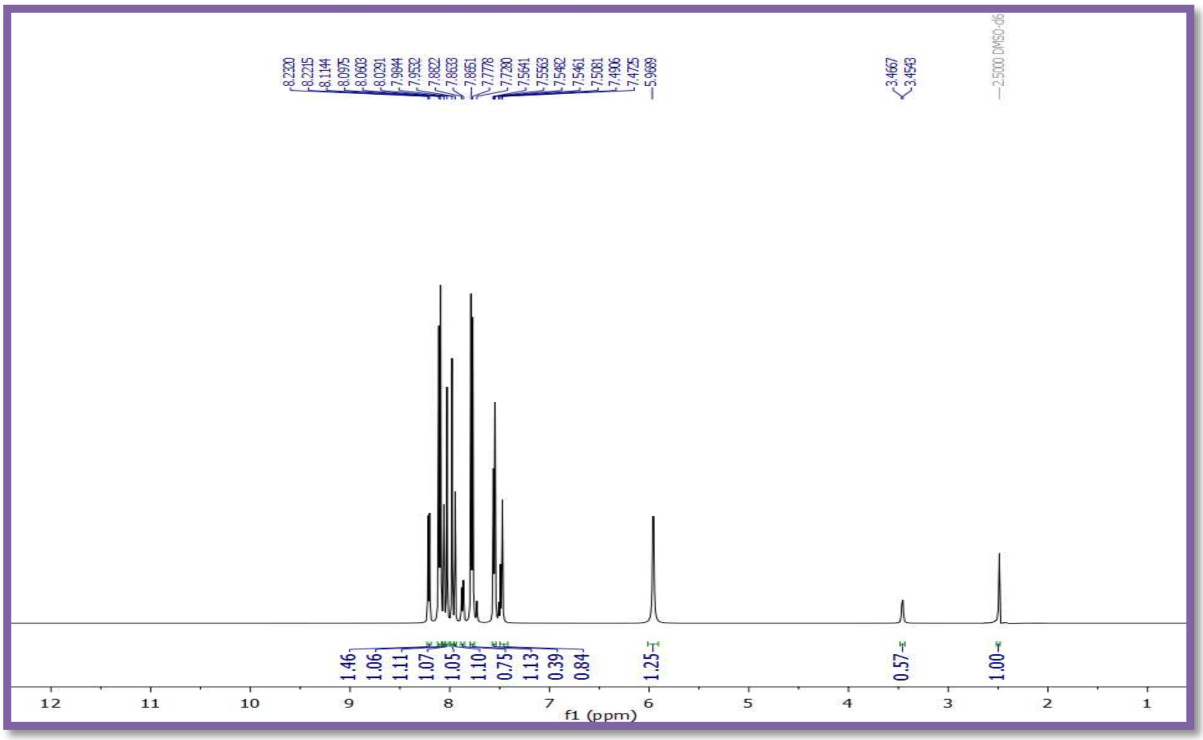

Figure S7. ^{1}H NMR spectrum of (2-(3-(4-bromophenyl)-5-(2-chlorophenyl)-1H-pyrazol-1-yl)-3,5-dinitrophenyl)tellurium tribromide (4).

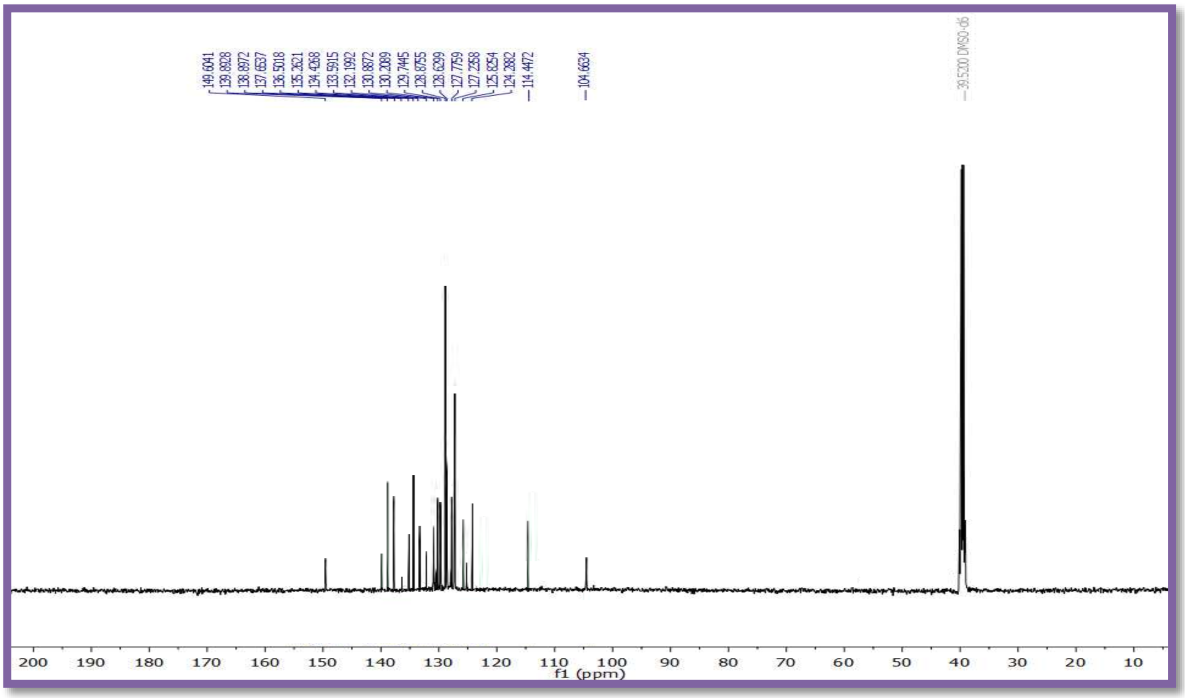

Figure S8. ^{13}C NMR spectrum of (2-(3-(4-bromophenyl)-5-(2-chlorophenyl)-1H-pyrazol-1-yl)-3,5-dinitrophenyl)tellurium tribromide (4).

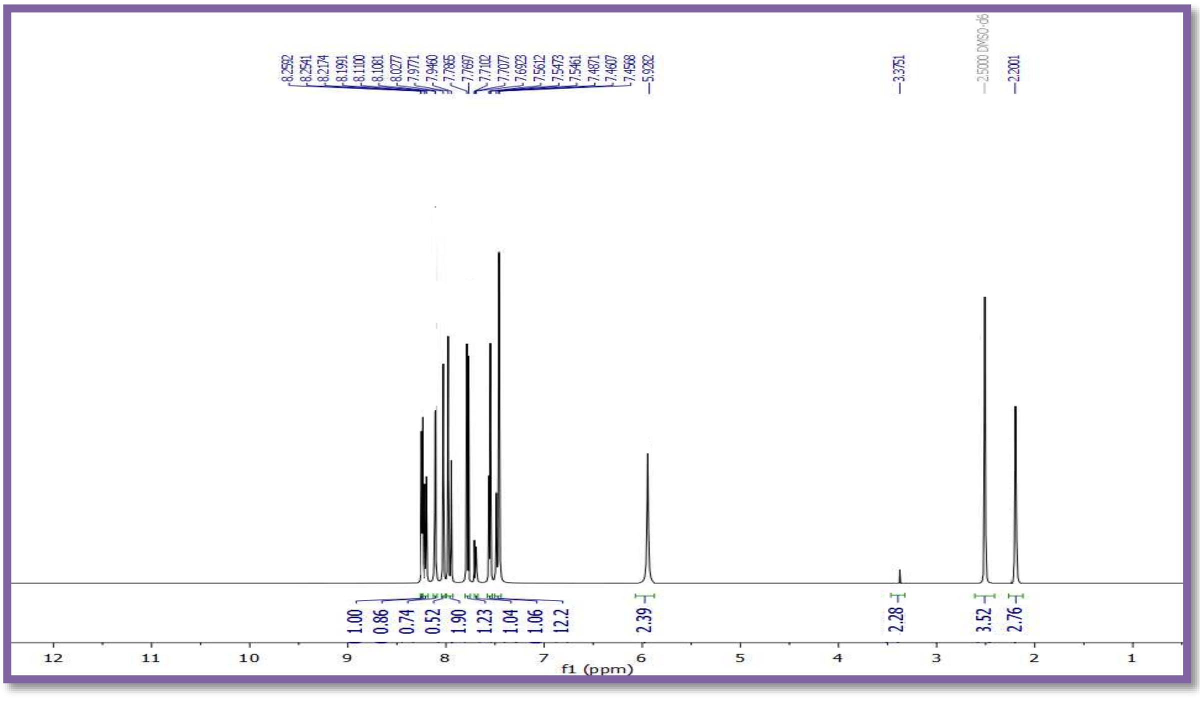

Figure S9. ^{1}H NMR spectrum of (2-(5-(2-chlorophenyl)-3-(4-methylphenyl)-1H-pyrazol-1-yl)-3,5-dinitrophenyl)tellurium tribromide (5).

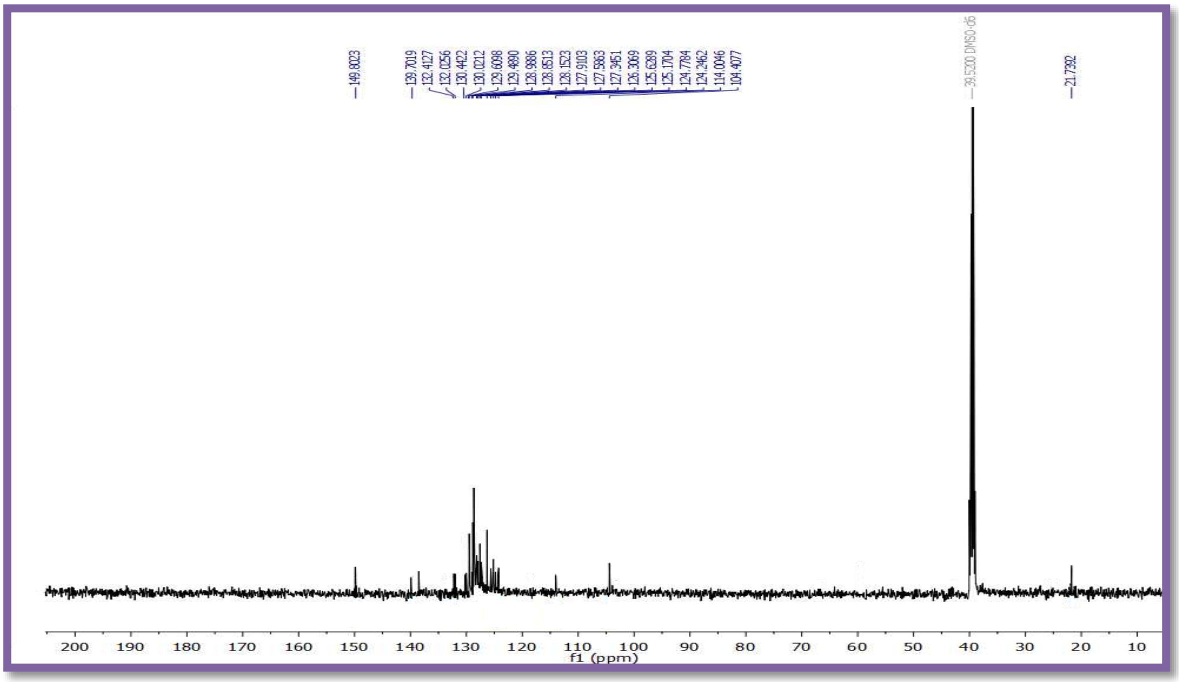

Figure S10. ^{13}C NMR spectrum of (2-(5-(2-chlorophenyl)-3-(4-methylphenyl)-1H-pyrazol-1-yl)-3,5-dinitrophenyl)tellurium tribromide (5).

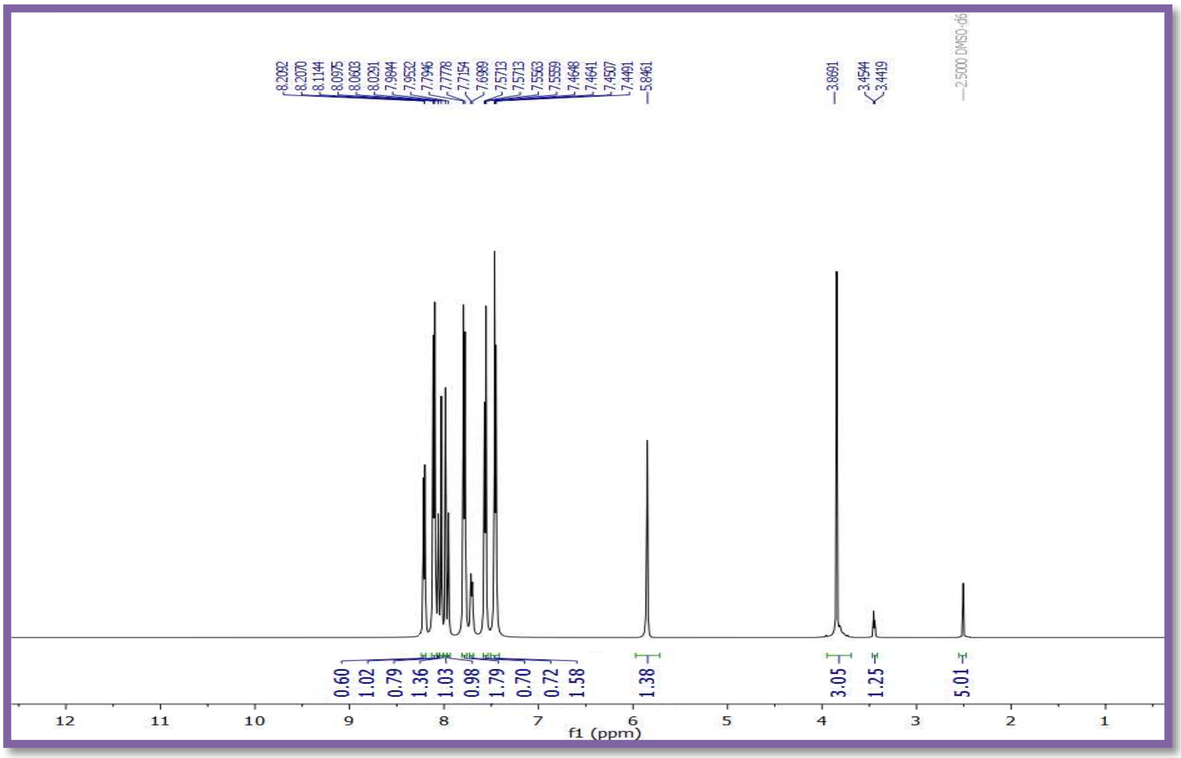

Figure S11. ^{1}H NMR spectrum of (2-(5-(2-chlorophenyl)-3-(4-methoxyphenyl)-1H-pyrazol-1-yl)-3,5dinitrophenyl)tellurium tribromide (6).

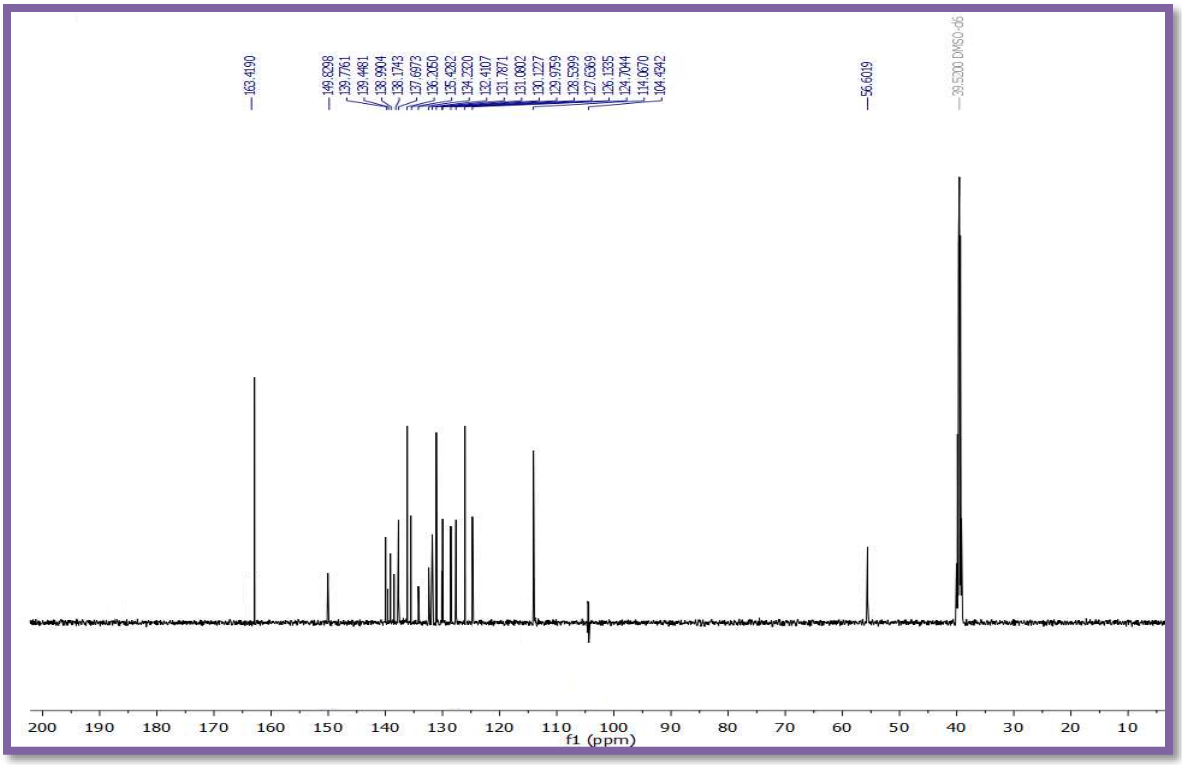

.

Figure S12. C NMR spectrum of (2-(5-(2-chlorophenyl)-3-(4-methoxyphenyl)-1H-pyrazol-1-yl)-3,5dinitrophenyl)tellurium tribromide (6).

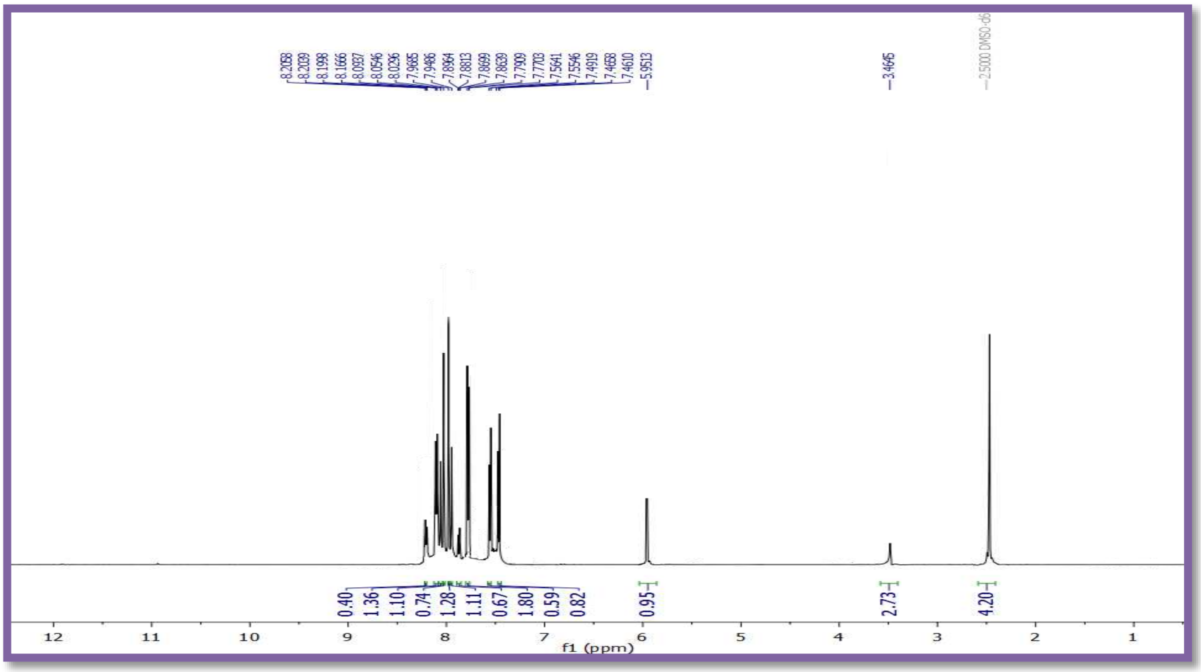

Figure S13: ^{1}H NMR spectrum of bis[(2-(3-(4-bromophenyl)-5-(2-chlorophenyl)-1H-pyrazol-1-yl)-3,5-dinitrophenyl)]tellurium dibromide (7).

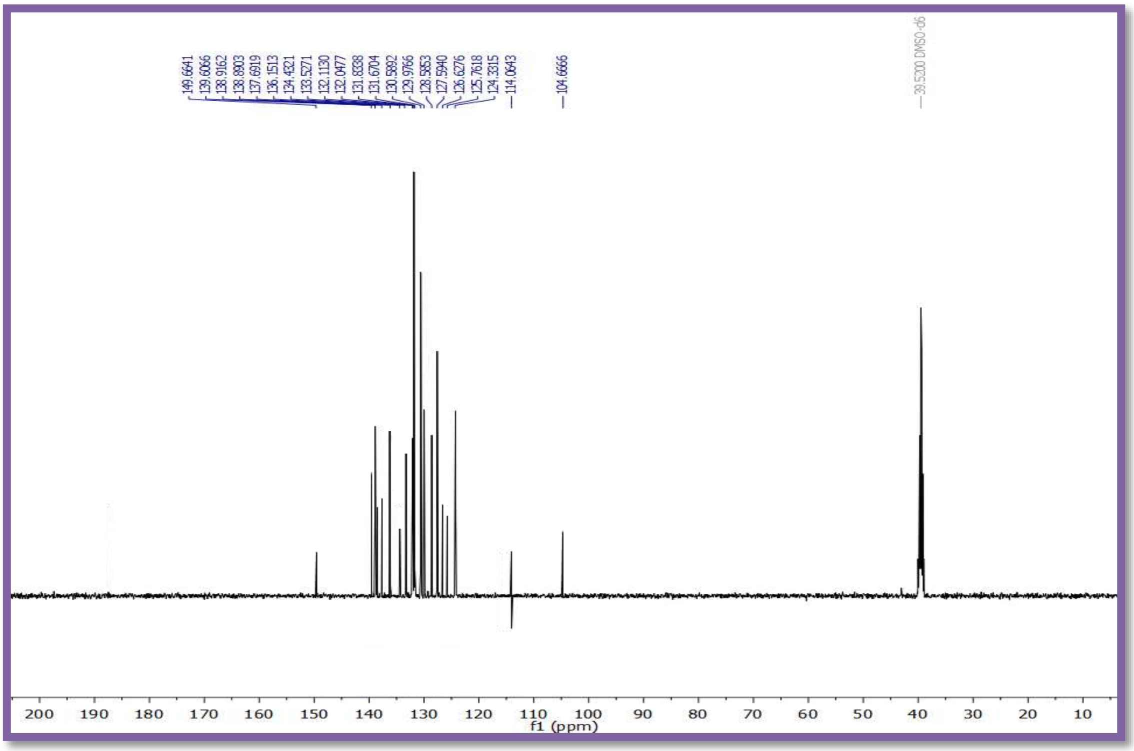

Figure S14. ^{13}C NMR spectrum of bis[(2-(3-(4-bromophenyl)-5-(2-chlorophenyl)-1H-pyrazol-1-yl)-3,5-dinitrophenyl)]tellurium dibromide (7).

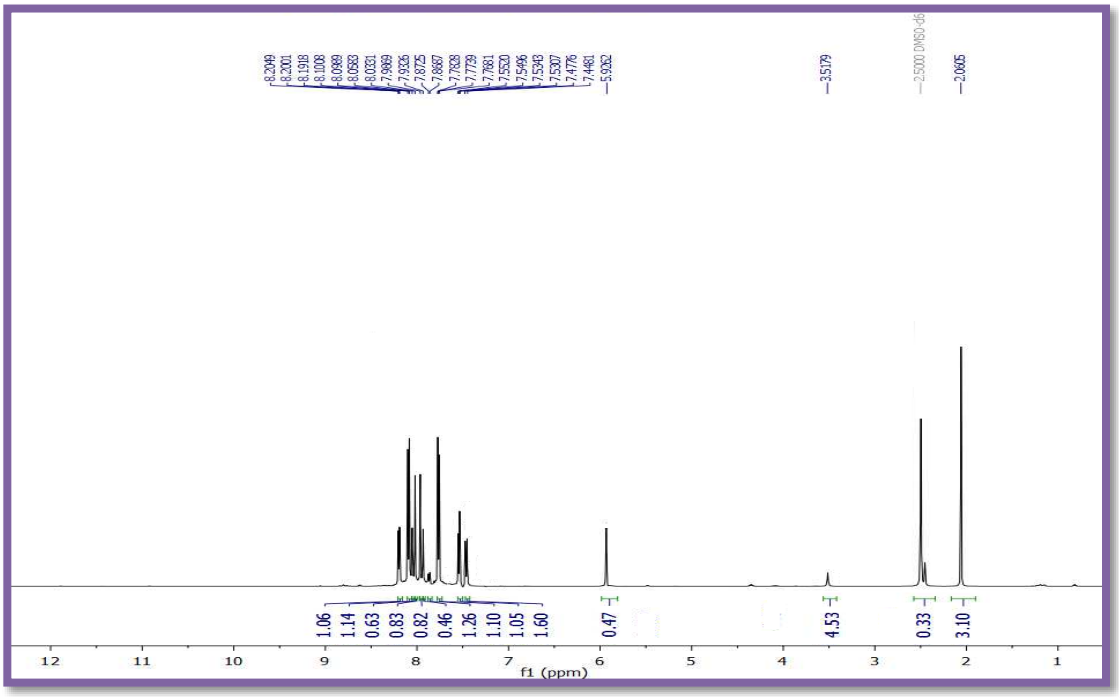

Figure S15. ^{1}H NMR spectrum of bis[(2-(5-(2-Chlorophenyl)-3-(4-methylphenyl)-1H-pyrazol-1-yl)-3,5-dinitrophenyl)]tellurium dibromide (8).

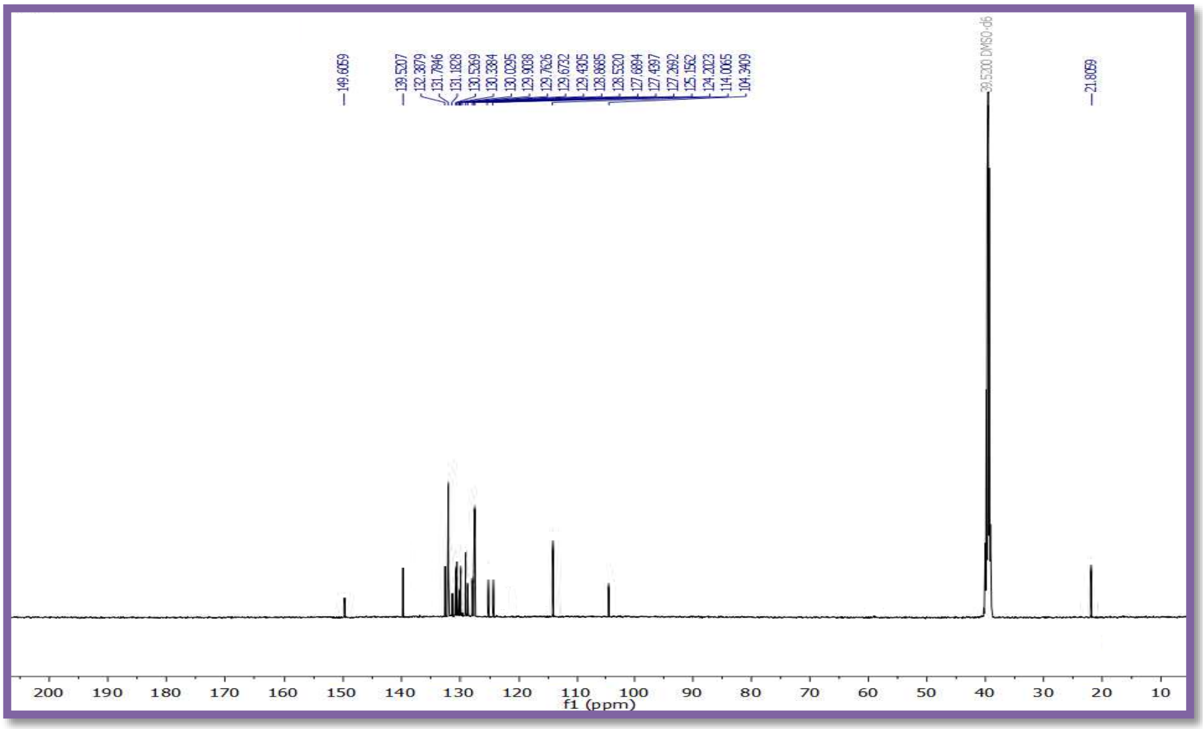

Figure S16. ^{13}C NMR spectrum of bis[(2-(5-(2-chlorophenyl)-3-(4-methylphenyl)-1H-pyrazol-1-yl)-3,5-dinitrophenyl)]tellurium dibromide (8).

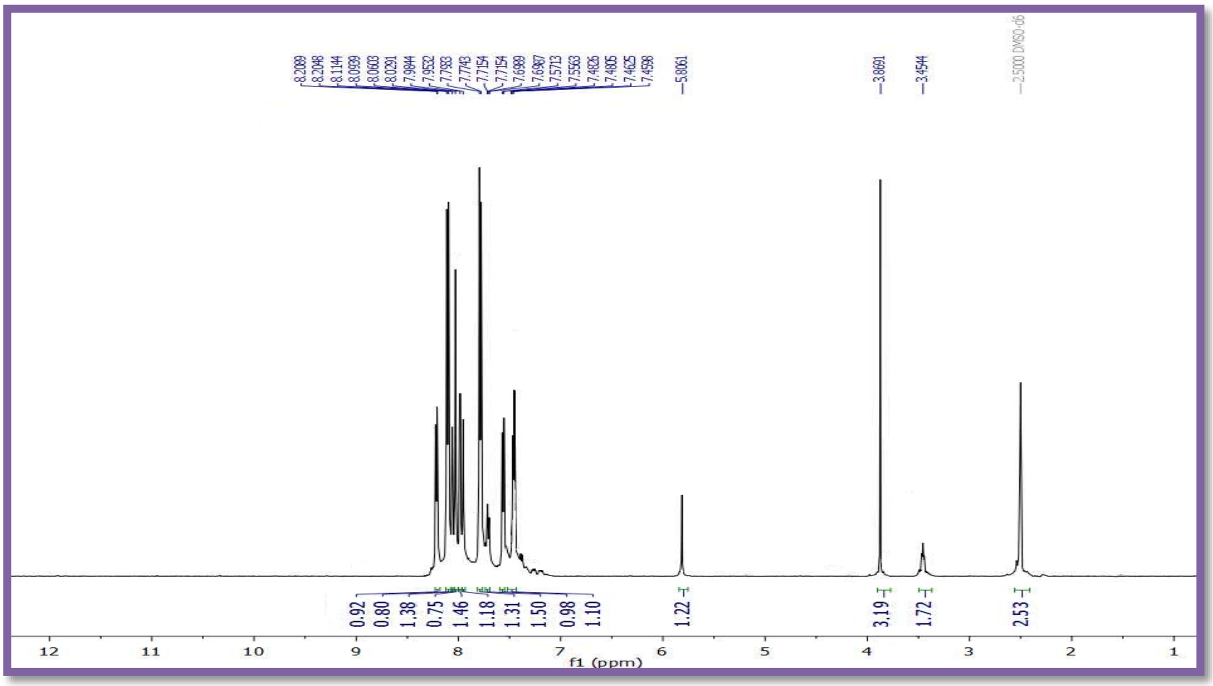

Figure S17. ^{1}H NMR spectrum of bis[2-(5-(2-chlorophenyl)-3-(4-methoxyphenyl)-1H-pyrazol-1-yl)-3,5dinitro phenyl)]tellurium dibromide (9).

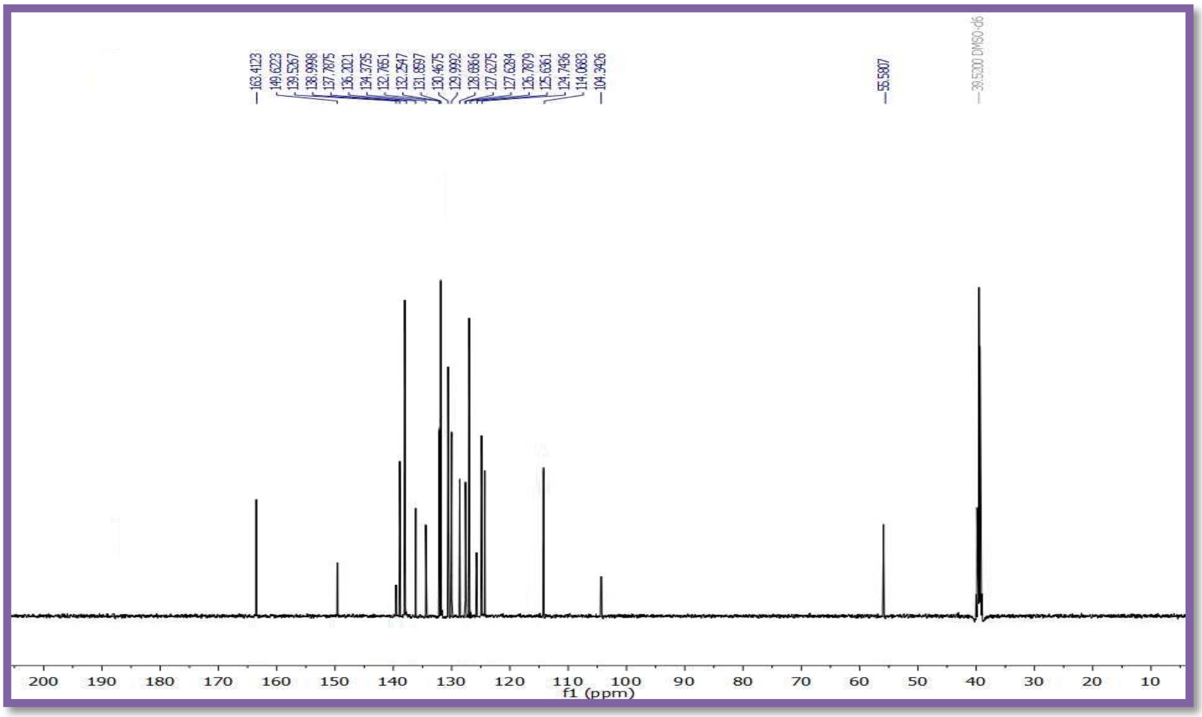

Figure S18. ^{13}C NMR spectrum of bis[2-(5-(2-chlorophenyl)-3-(4-methoxyphenyl)-1H-pyrazol-1-yl)-3,5dinitro phenyl)]tellurium dibromide (9).

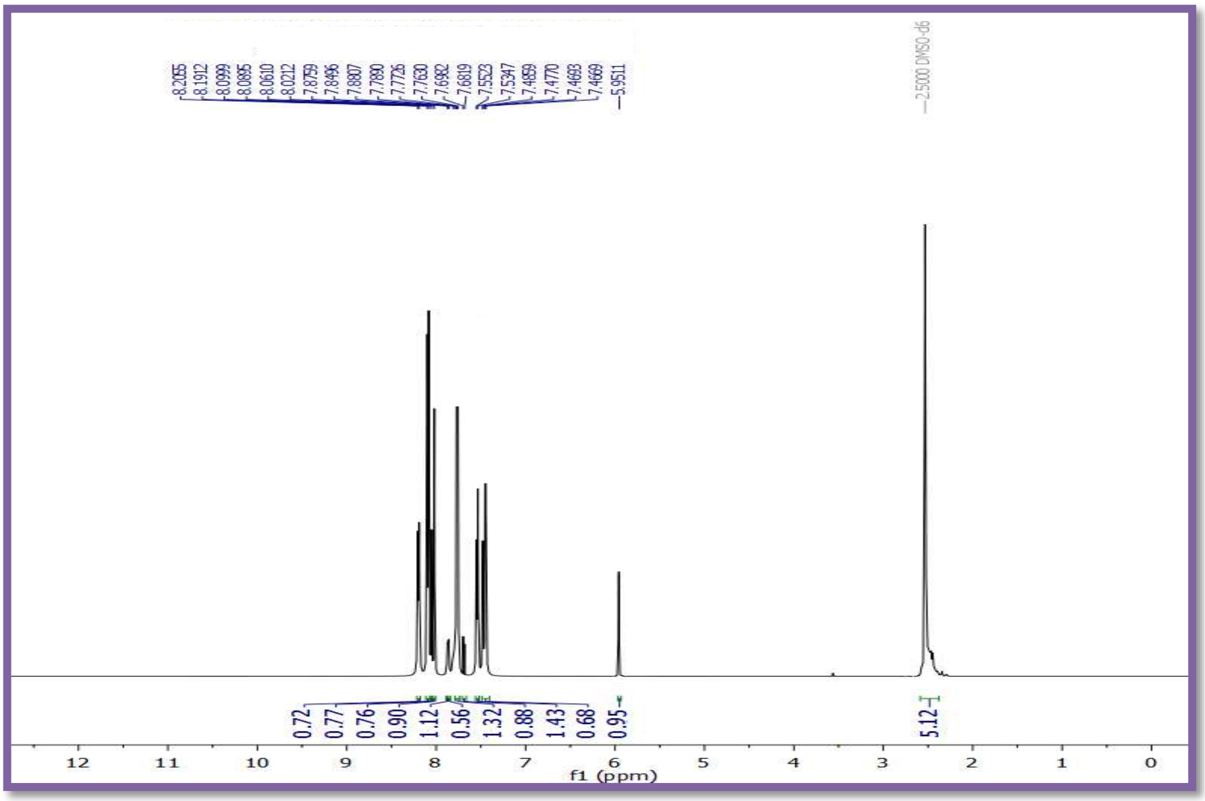

Figure S19. ^{1}H NMR spectrum of bis[(2-(3-(4-bromophenyl)-5-(2-chlorophenyl)-1H-pyrazol-1-yl)-3,5-dinitrophenyl)] ditelluride (10).

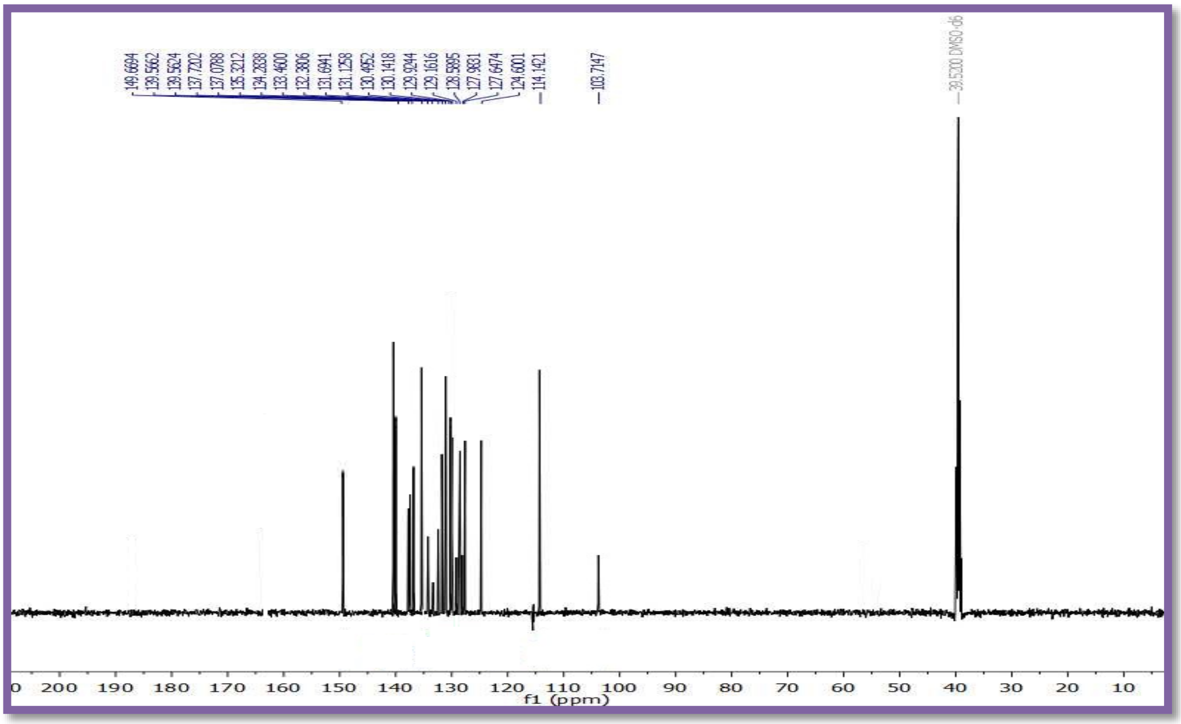

Figure S20. ^{13}C NMR spectrum of bis[(2-(3-(4-bromophenyl)-5-(2-chlorophenyl)-1H-pyrazol-1-yl)-3,5-dinitrophenyl)] ditelluride (10).

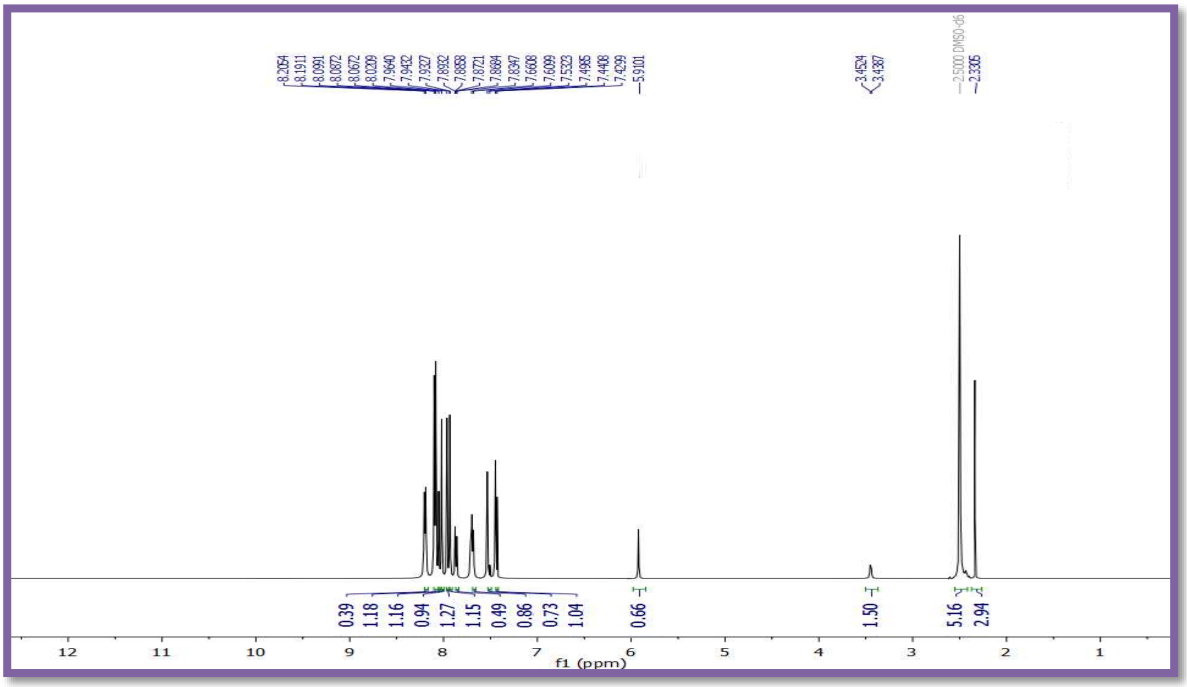

Figure S21. ^{1}H NMR spectrum of bis[(2-(5-(2-chlorophenyl)-3-(4-methylphenyl)-1H-pyrazol-1-yl)-3,5-dinitrophenyl)] ditelluride (11).

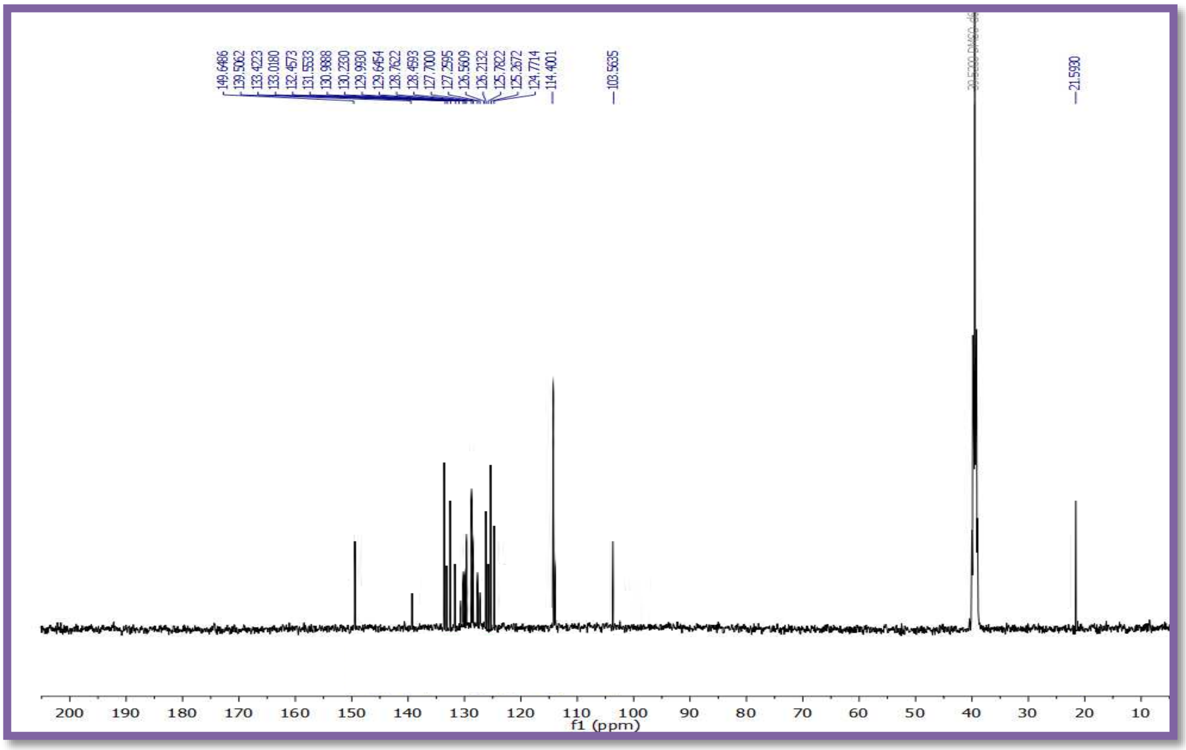

Figure S22. ^{13}C NMR spectrum of bis[(2-(5-(2-chlorophenyl)-3-(4-methylphenyl)-1H-pyrazol-1-yl)-3,5-dinitrophenyl)] ditelluride (11).

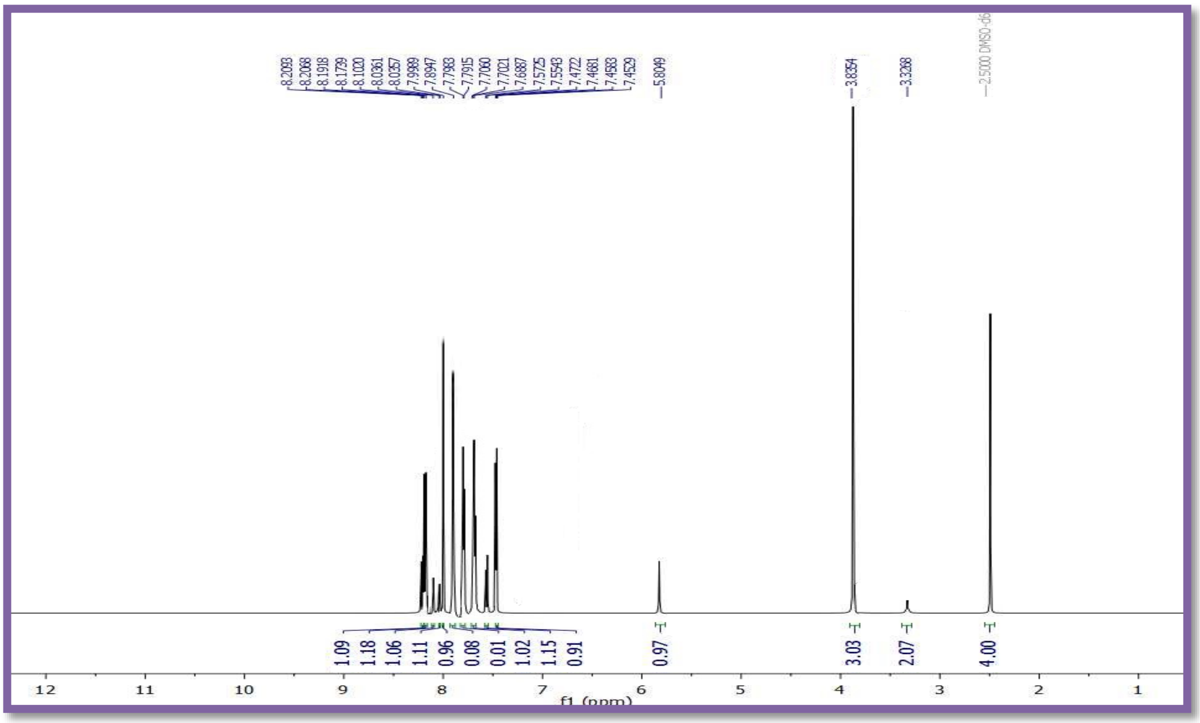

Figure S23. ^{1}H NMR spectrum of bis[(2-(5-(2-chlorophenyl)-3-(4-methoxyphenyl)-1H-pyrazol-1-yl)-3,5dinitro phenyl)] ditelluride (12).

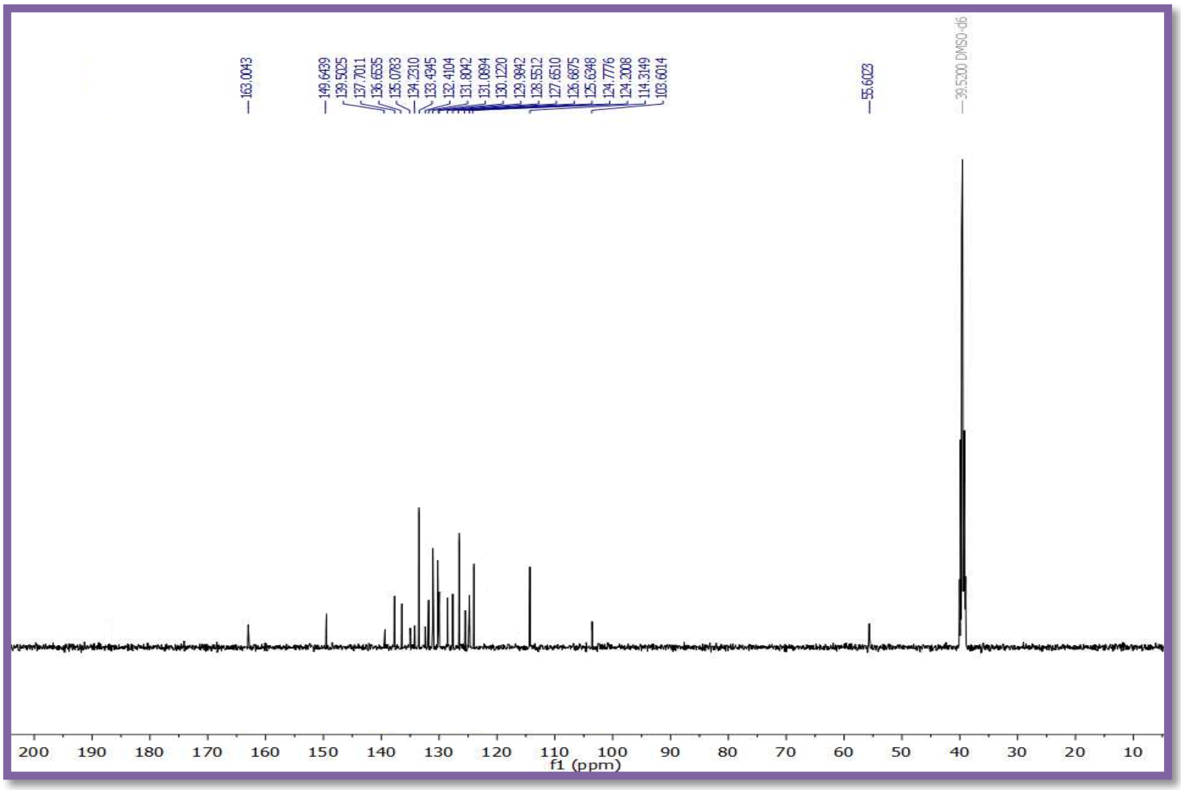

Figure S24. ^{13}C NMR spectrum of bis[(2-(5-(2-chlorophenyl)-3-(4-methoxyphenyl)-1H-pyrazol-1-yl)-3,5dinitro phenyl)] ditelluride (12).

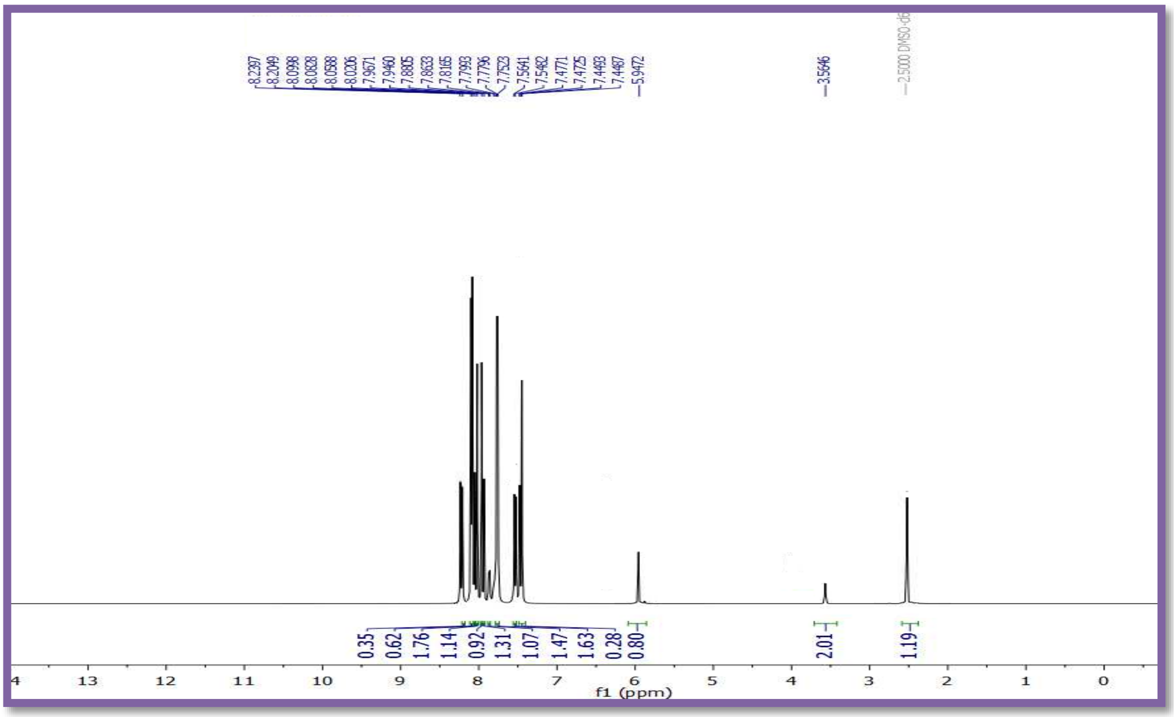

Figure S25. ^{1}H NMR spectrum of bis[(2-(3-(4-bromophenyl)-5-(2-chlorophenyl)-1H-pyrazol-1-yl)-3,5-dinitrophenyl)] telluride (13).

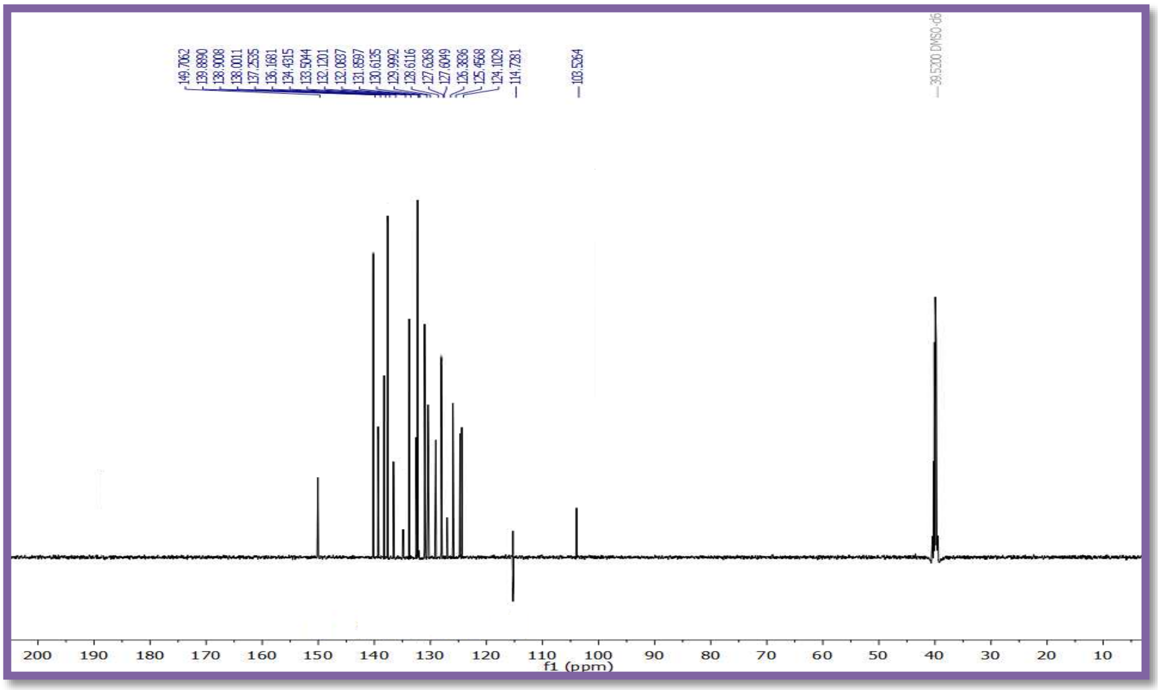

Figure S26. C NMR spectrum of bis[(2-(3-(4-bromophenyl)-5-(2-chlorophenyl)-1H-pyrazol-1-yl)-3,5-dinitrophenyl)] telluride (13).

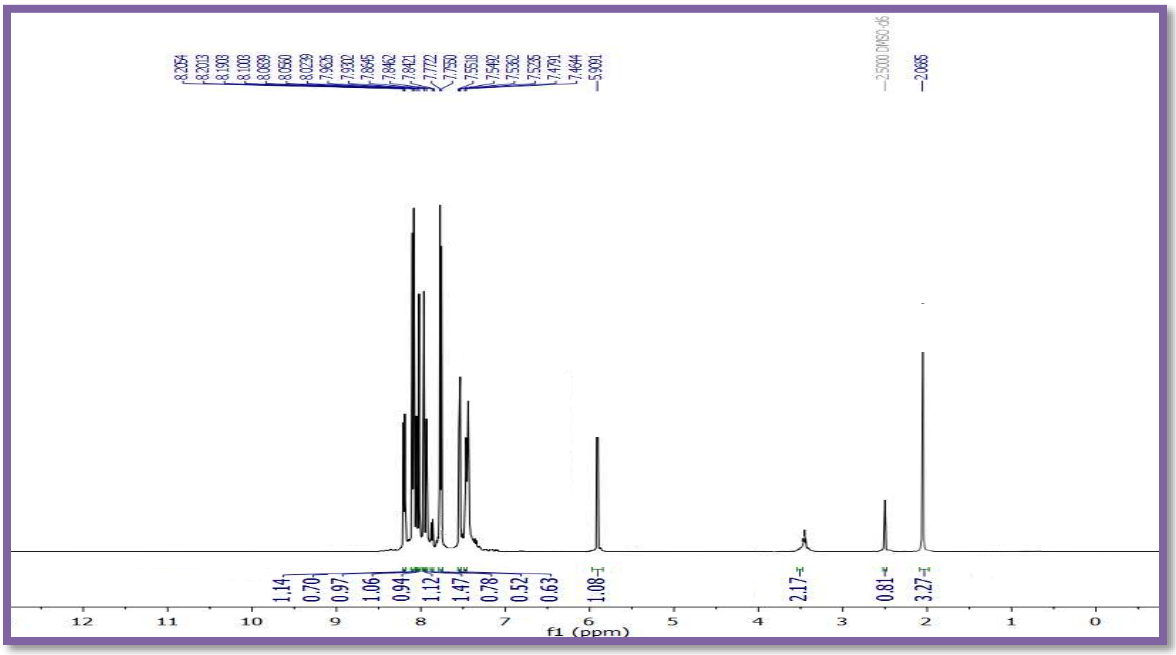

Figure S27. 1H NMR spectrum of bis[(2-(5-(2-chlorophenyl)-3-(4-methylphenyl)-1H-pyrazol-1-yl)-3,5-dinitrophenyl)] telluride (14).

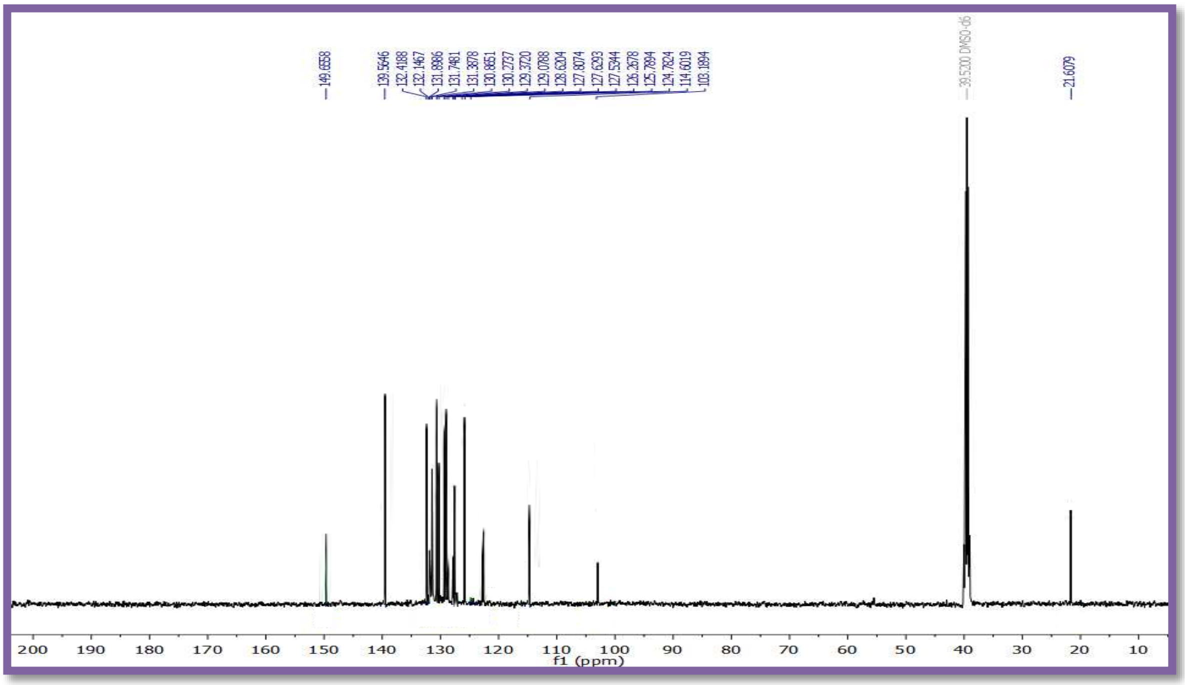

Figure S28. ^{13}C NMR spectrum of bis[(2-(5-(2-chlorophenyl)-3-(4-methylphenyl)-1H-pyrazol-1-yl)-3,5-dinitrophenyl)] telluride (14).

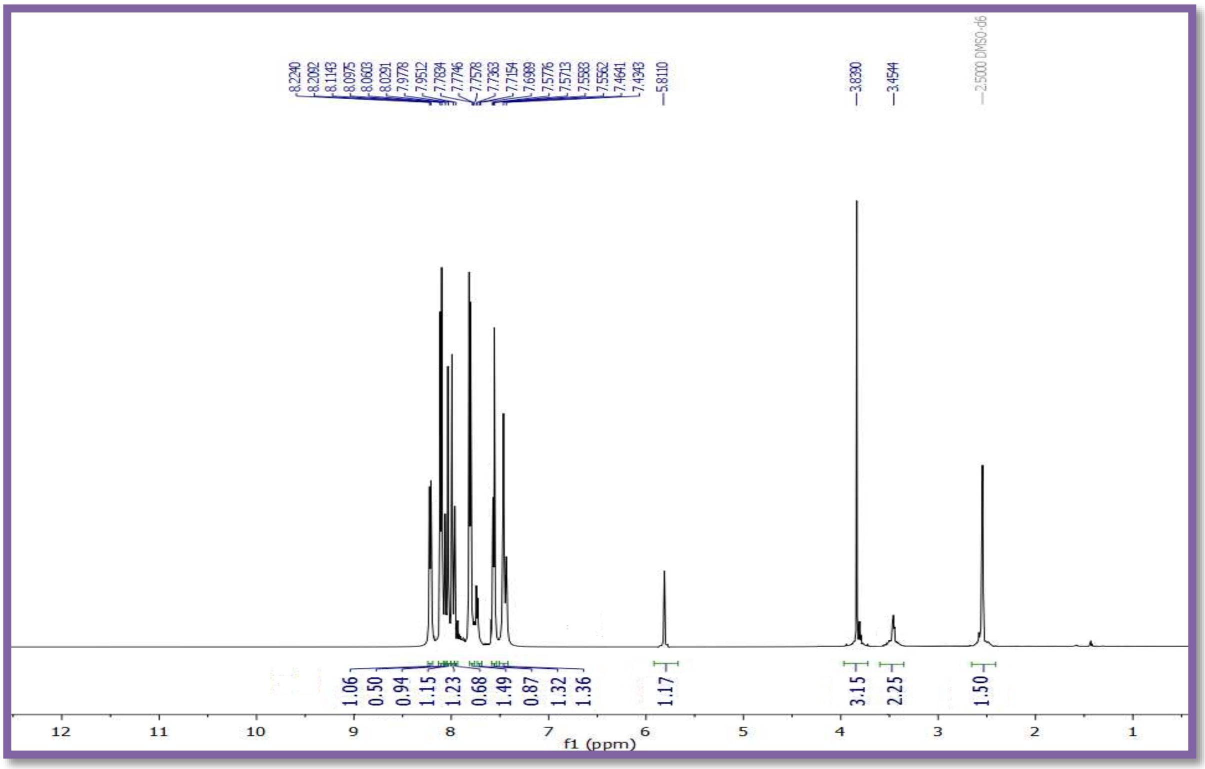

Figure S29. ^{1}H NMR spectrum of bis[(2-(5-(2-chlorophenyl)-3-(4-methoxyphenyl)-1H-pyrazol-1-yl)-3,5-dinitrophenyl)] telluride (15).

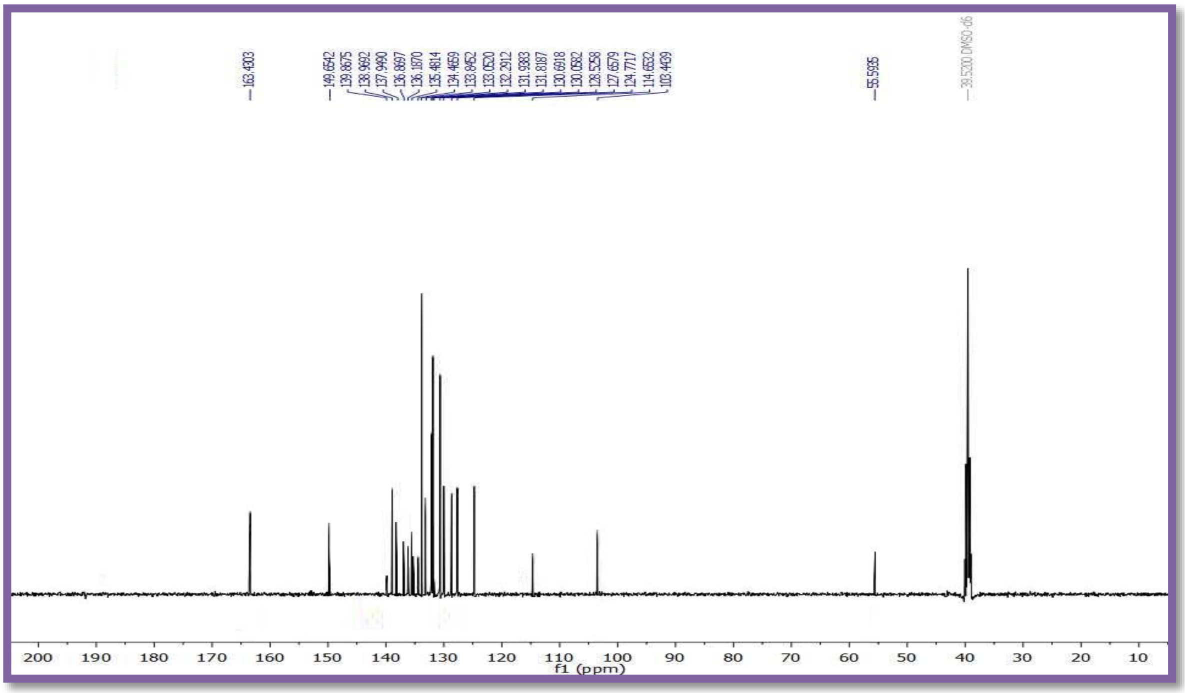

Figure S30. ^{13}C NMR spectrum of bis[(2-(5-(2-chlorophenyl)-3-(4-methoxyphenyl)-1H-pyrazol-1-yl)-3,5-dinitrophenyl)] telluride (15).

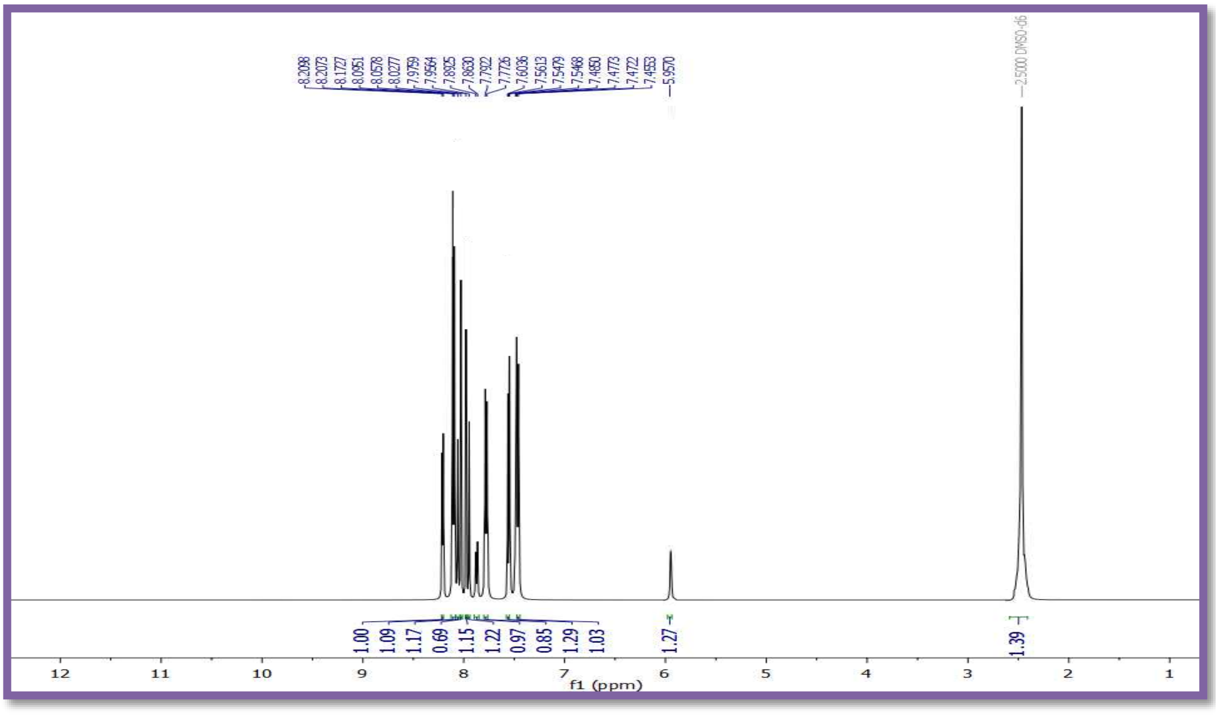

Figure S31. ^{1}H NMR spectrum of (2-(3-(4-bromophenyl)-5-(2-chlorophenyl)-1H-pyrazol-1-yl)-3,5-dinitrophenyl)tellurium trichloride (16).

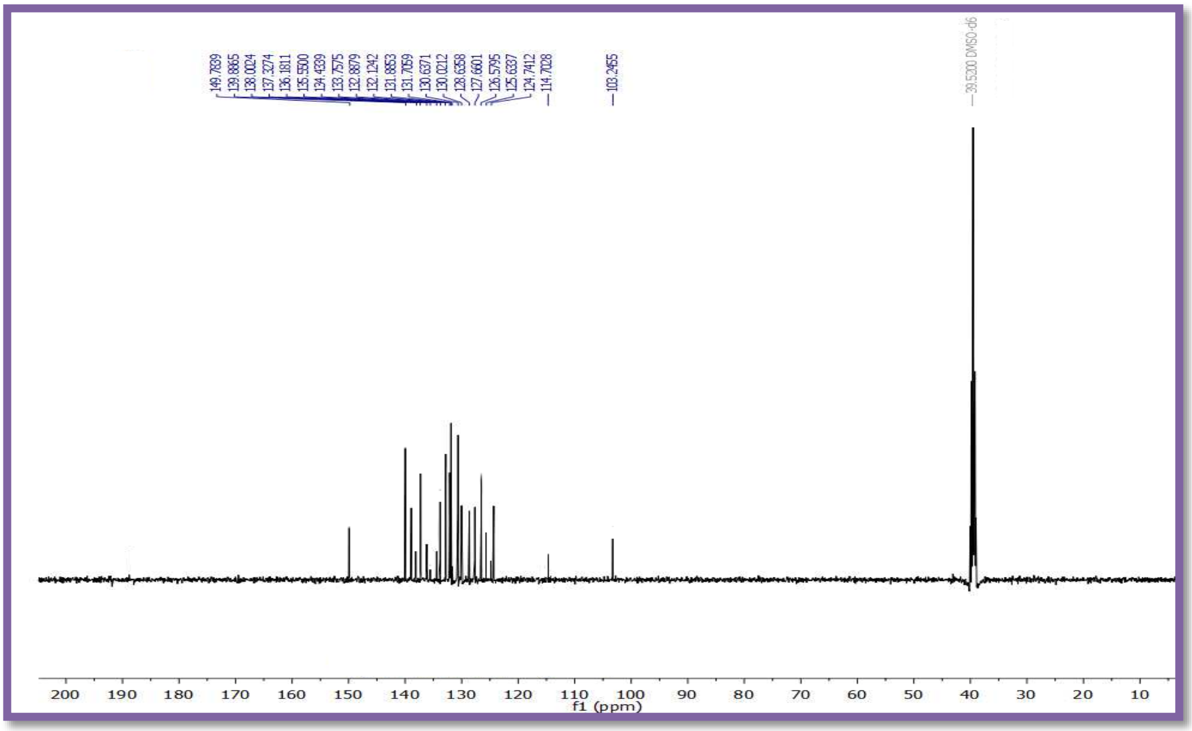

Figure S32. ^{13}C NMR spectrum of (2-(3-(4-bromophenyl)-5-(2-chlorophenyl)-1H-pyrazol-1-yl)-3,5-dinitrophenyl)tellurium trichloride (16).

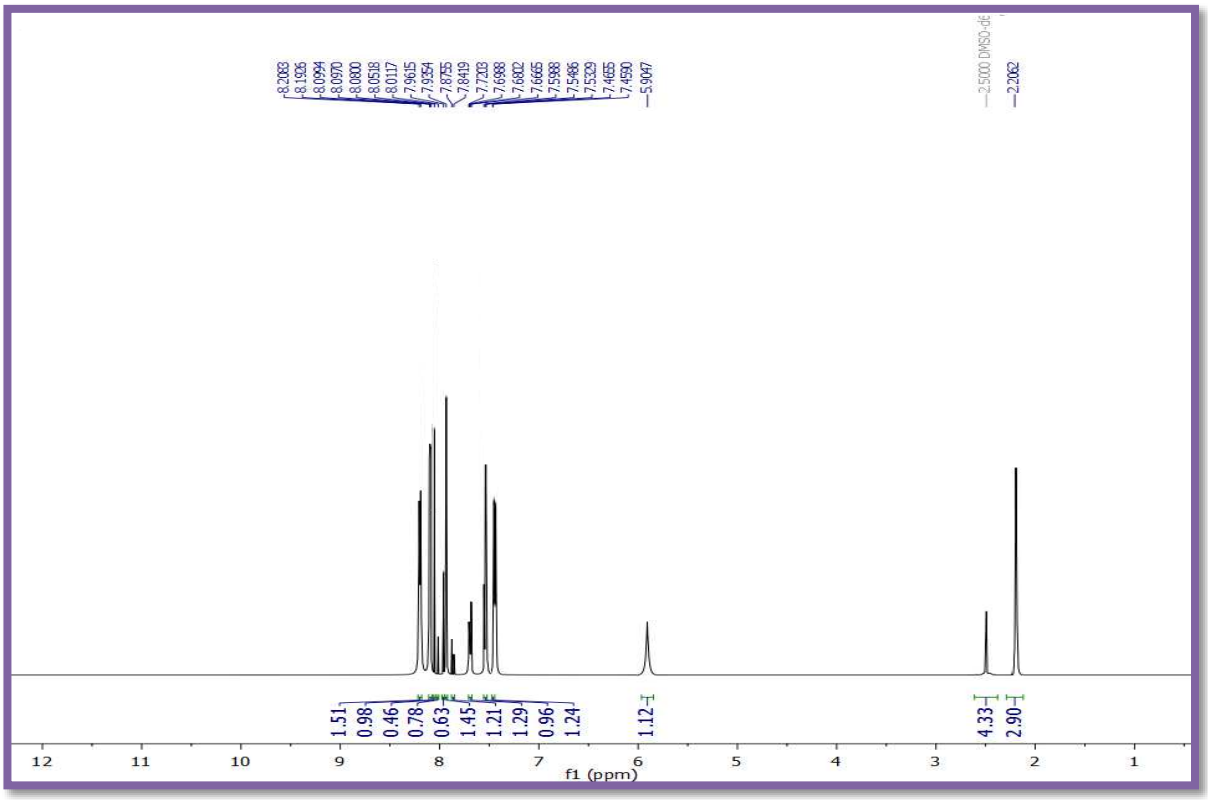

Figure S33. ^{1}H NMR spectrum of (2-(3-(4-methylphenyl)-5-(2-chlorophenyl)-1H-pyrazol-1-yl)-3,5-dinitrophenyl)tellurium trichloride (17).

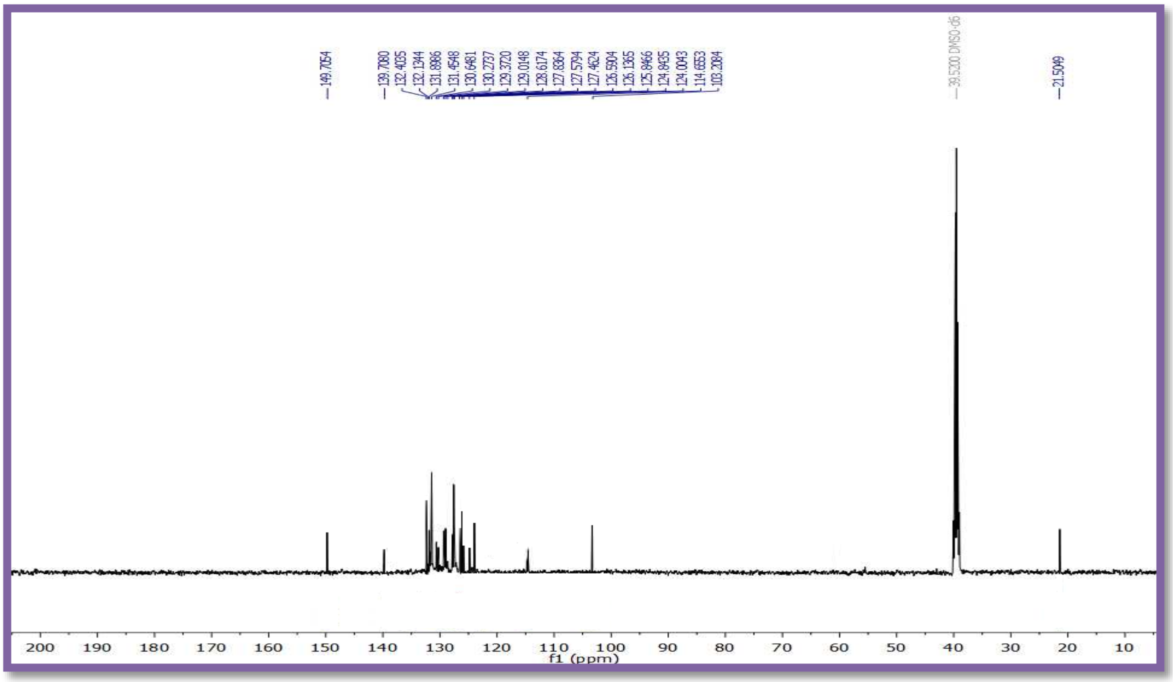

Figure S34. ^{13}C NMR spectrum of (2-(3-(4-methylphenyl)-5-(2-chlorophenyl)-1H-pyrazol-1-yl)-3,5-dinitrophenyl)tellurium trichloride (17).

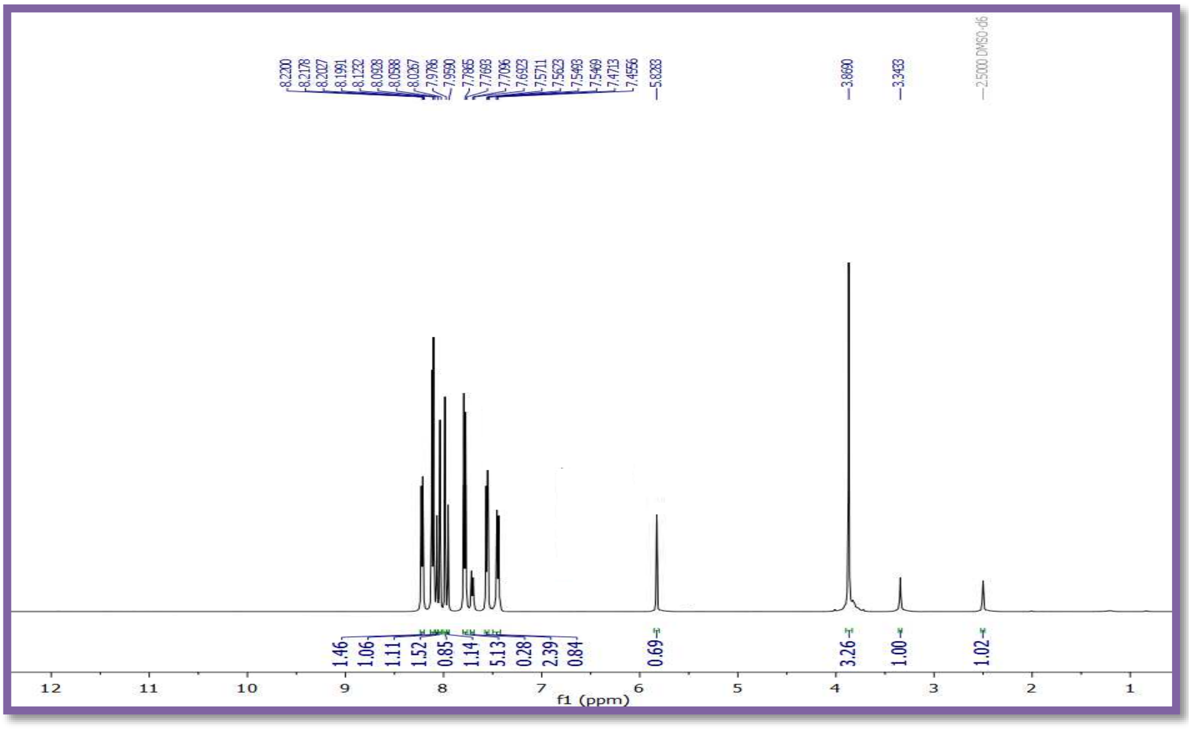

Figure S35. ^{1}H NMR spectrum of (2-(3-(4-methoxyphenyl)-5-(2-chlorophenyl)-1H-pyrazol-1-yl)-3,5-dinitrophenyl)tellurium trichloride (18).

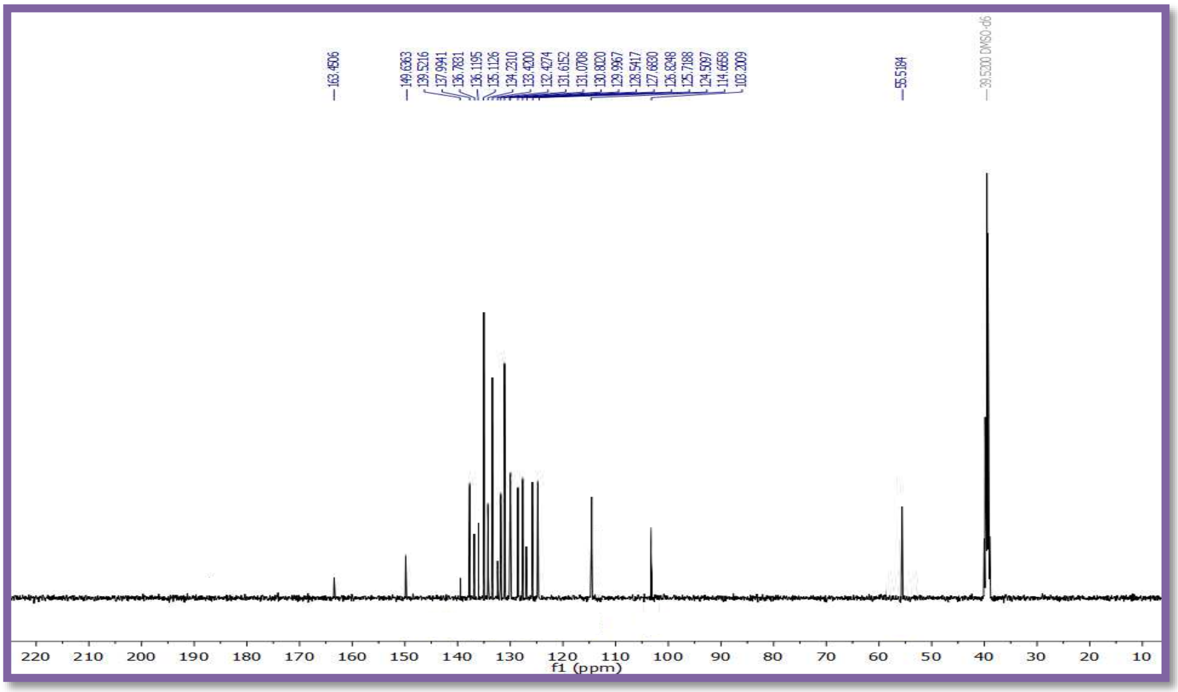

Figure S36. ^{13}C NMR spectrum of (2-(3-(4-methoxyphenyl)-5-(2-chlorophenyl)-1H-pyrazol-1-yl)-3,5-dinitrophenyl)tellurium trichloride (18).

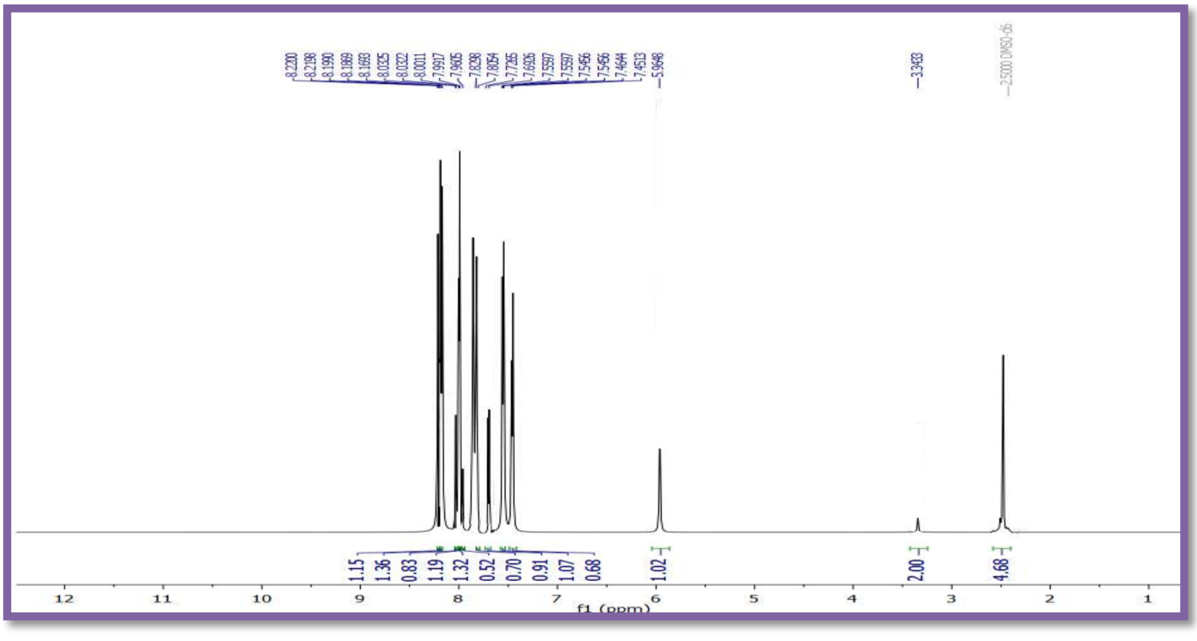

Figure S37. ^{1}H NMR spectrum of (2-(3-(4-bromophenyl)-5-(2-chlorophenyl)-1*H*-pyrazol-1-yl)-3,5-dinitrophenyl)tellurium triiodide (19).

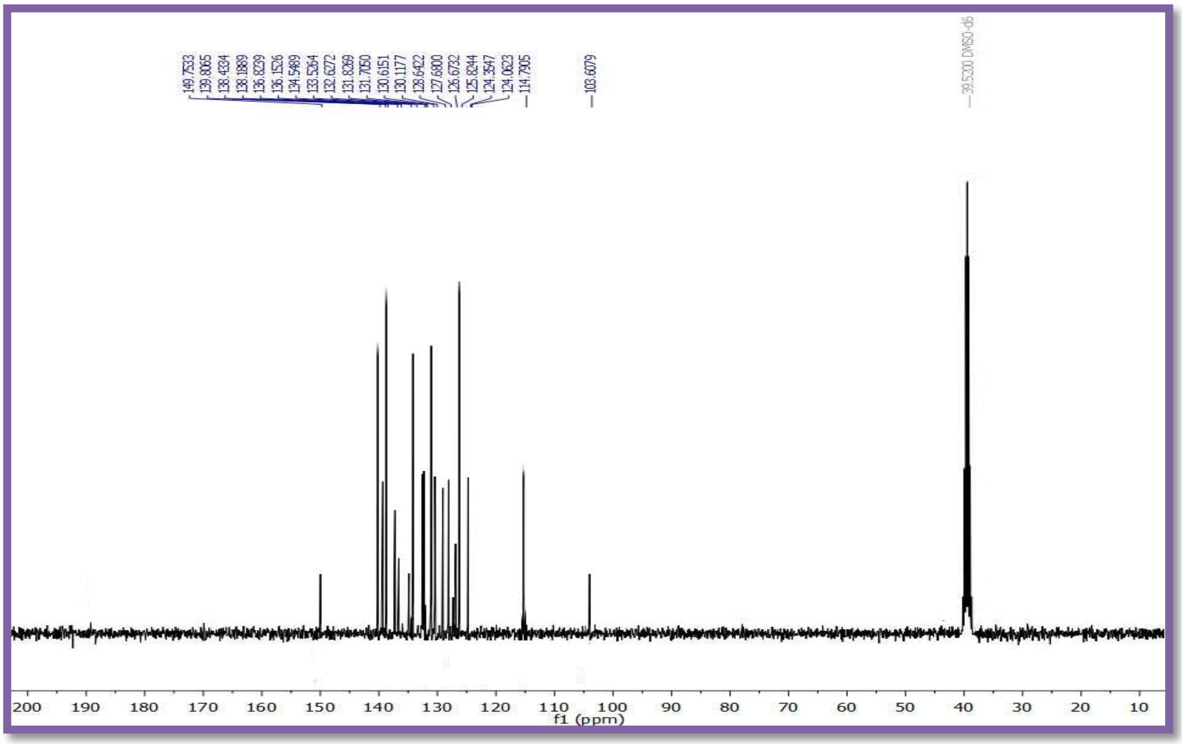

Figure S38. C NMR spectrum of (2-(3-(4-bromophenyl)-5-(2-chlorophenyl)-1*H*-pyrazol-1-yl)-3,5-dinitrophenyl)tellurium triiodide (19).

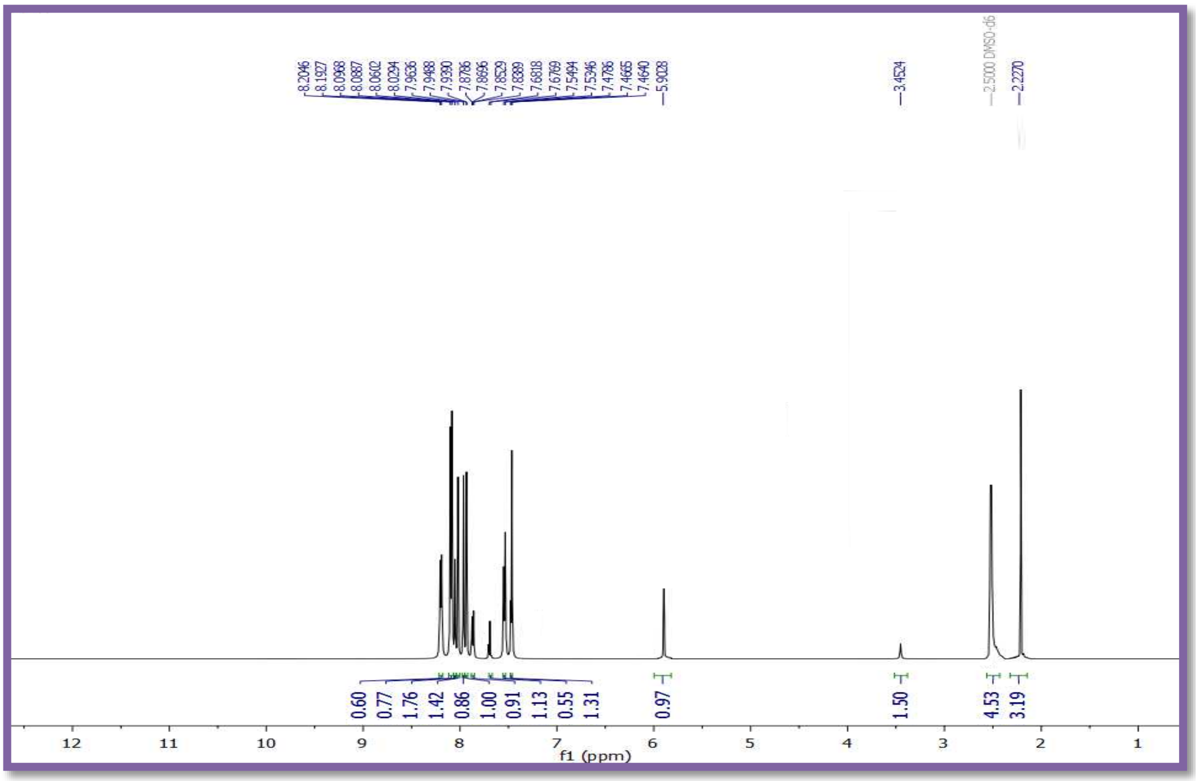

Figure S39. ^{1}H NMR spectrum of (2-(3-(4-methylphenyl)-5-(2-chlorophenyl)-1*H*-pyrazol-1-yl)-3,5-dinitrophenyl)tellurium triiodide (20).

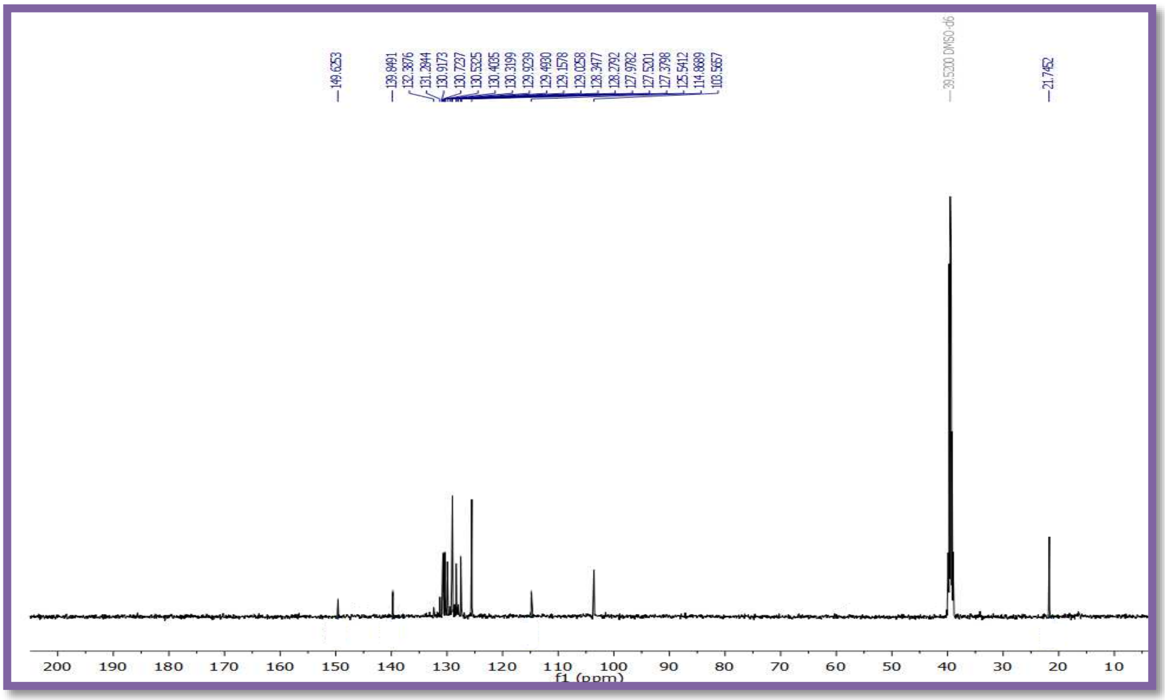

Figure S40. ^{13}C NMR spectrum of (2-(3-(4-methylphenyl)-5-(2-chlorophenyl)-1*H*-pyrazol-1-yl)-3,5-dinitrophenyl)tellurium triiodide (20).

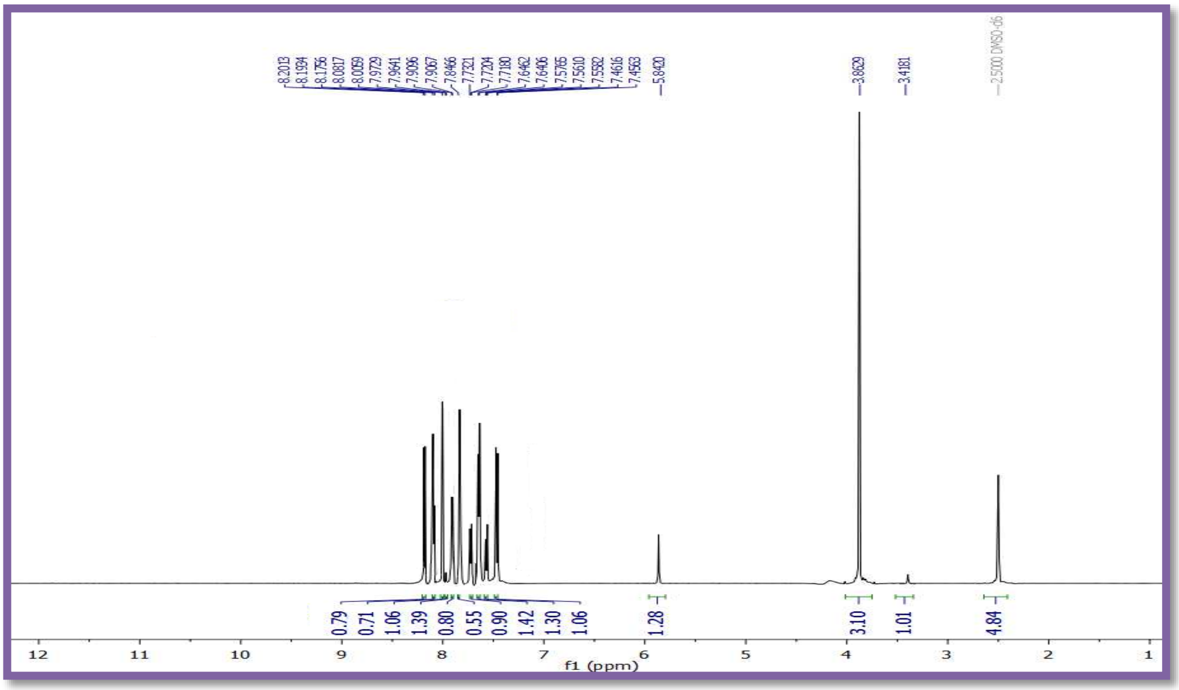

Figure S41. ^{1}H NMR spectrum of (2-(3-(4-methoxyphenyl)-5-(2-chlorophenyl)-1*H*-pyrazol-1-yl)-3,5-dinitrophenyl)tellurium triiodide (21).

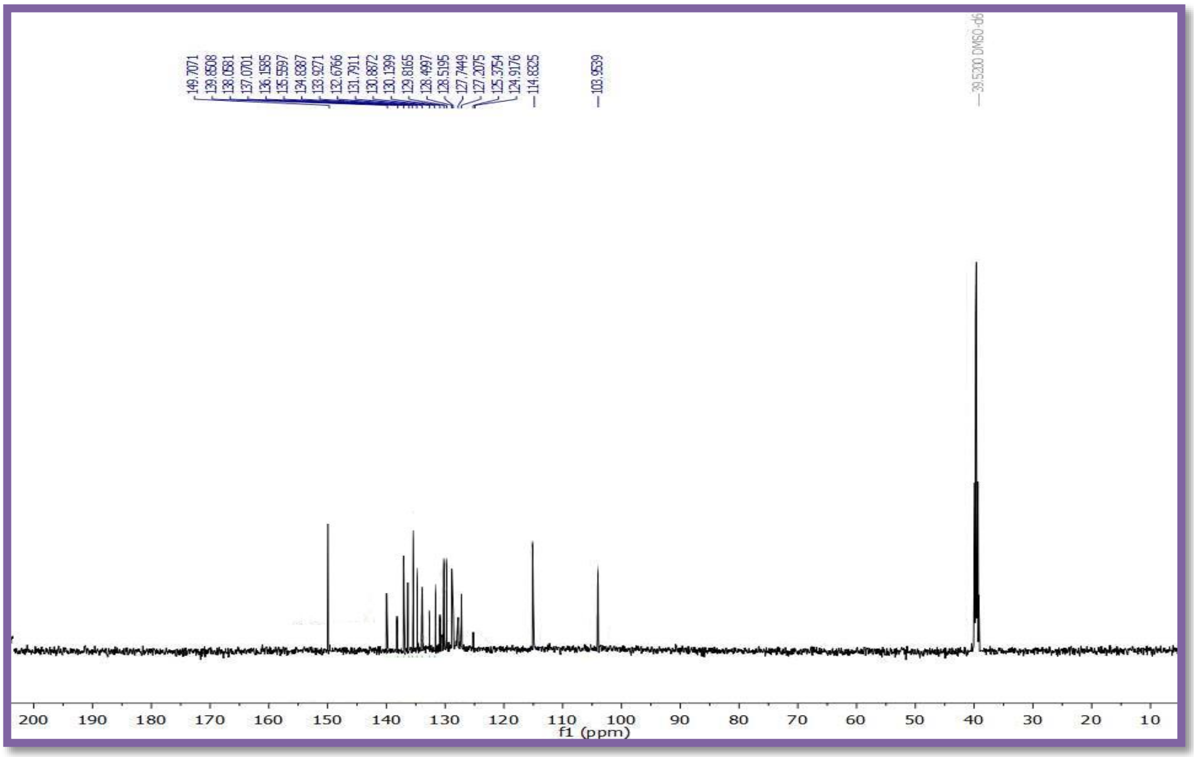

Figure S42. ^{13}C NMR spectrum of (2-(3-(4-methoxyphenyl)-5-(2-chlorophenyl)-1*H*-pyrazol-1-yl)-3,5-dinitrophenyl)tellurium triiodide (21).

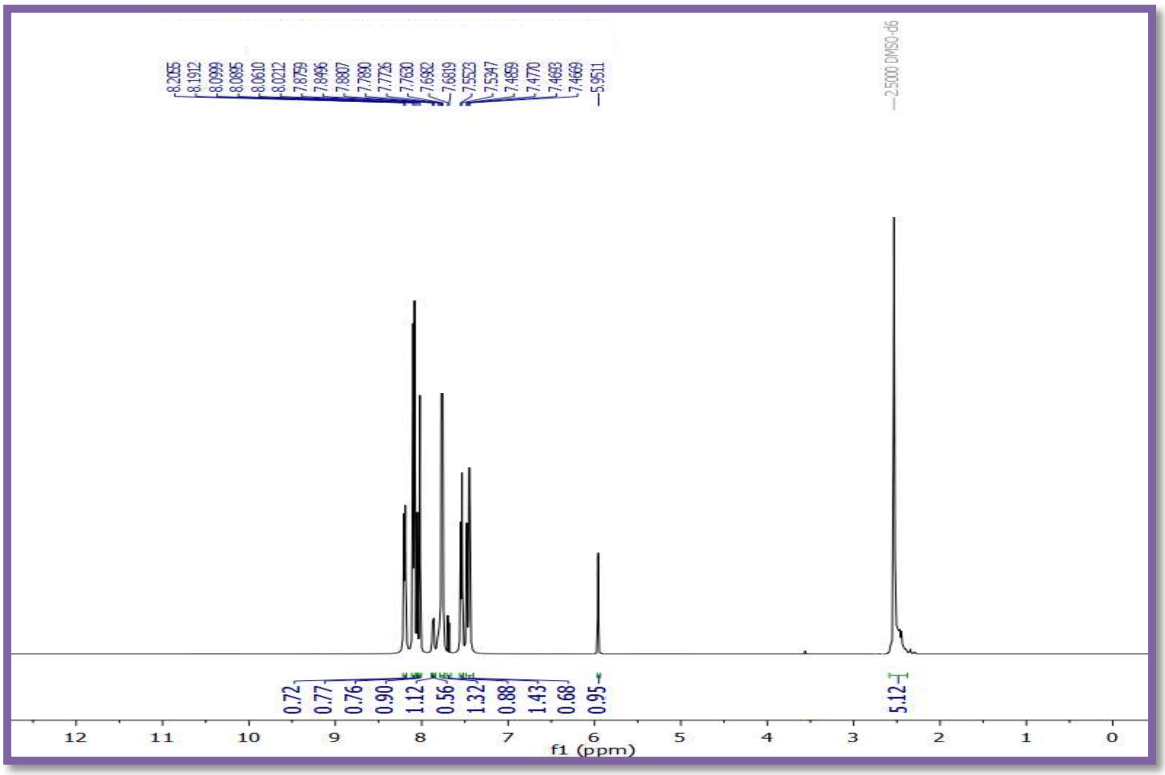

Figure S43. ^{1}H NMR spectrum of bis[(2-(3-(4-bromophenyl)-5-(2-chlorophenyl)-1H-pyrazol-1-yl)-3,5-dinitrophenyl)]tellurium dichloride (22).

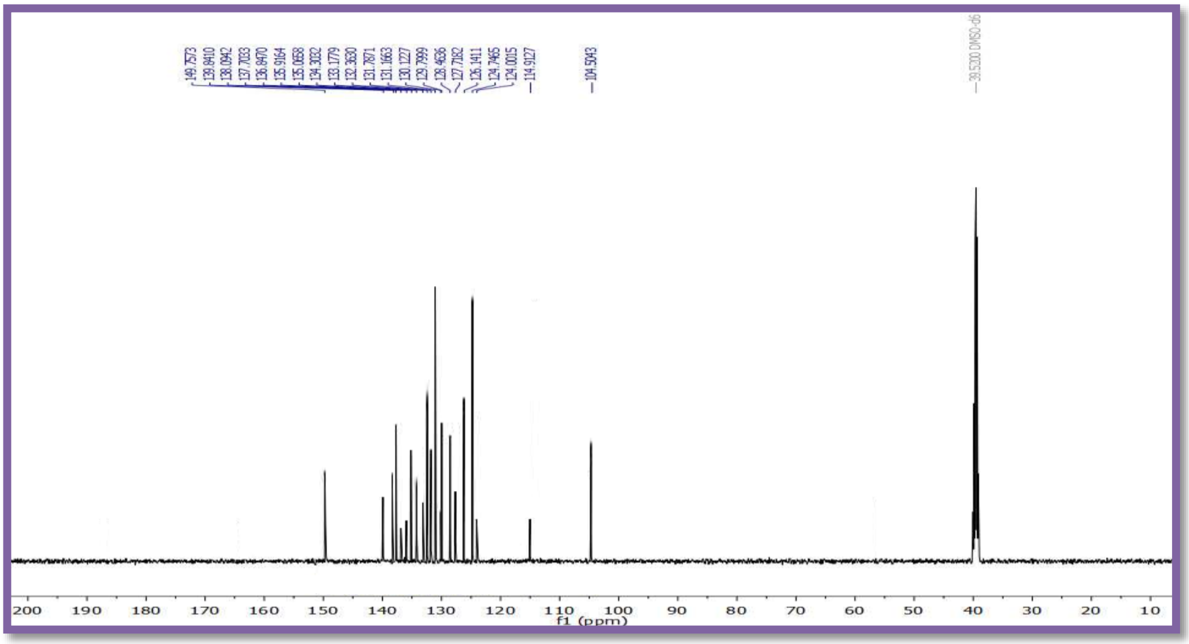

Figure S44. ^{13}C NMR spectrum of bis[(2-(3-(4-bromophenyl)-5-(2-chlorophenyl)-1H-pyrazol-1-yl)-3,5-dinitrophenyl)]tellurium dichloride (22).

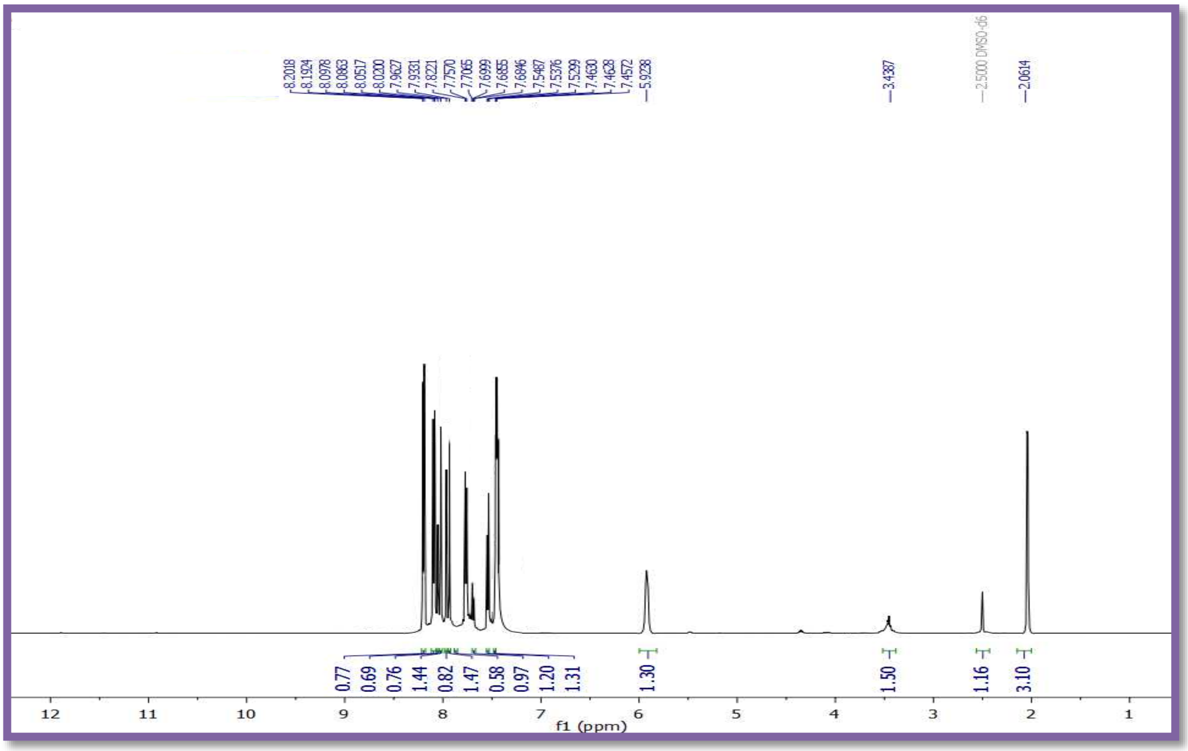

Figure S45. ^{1}H NMR spectrum of bis[(2-(3-(4-methylphenyl)-5-(2-chlorophenyl)-1H-pyrazol-1-yl)-3,5-dinitrophenyl)]tellurium dichloride (23).

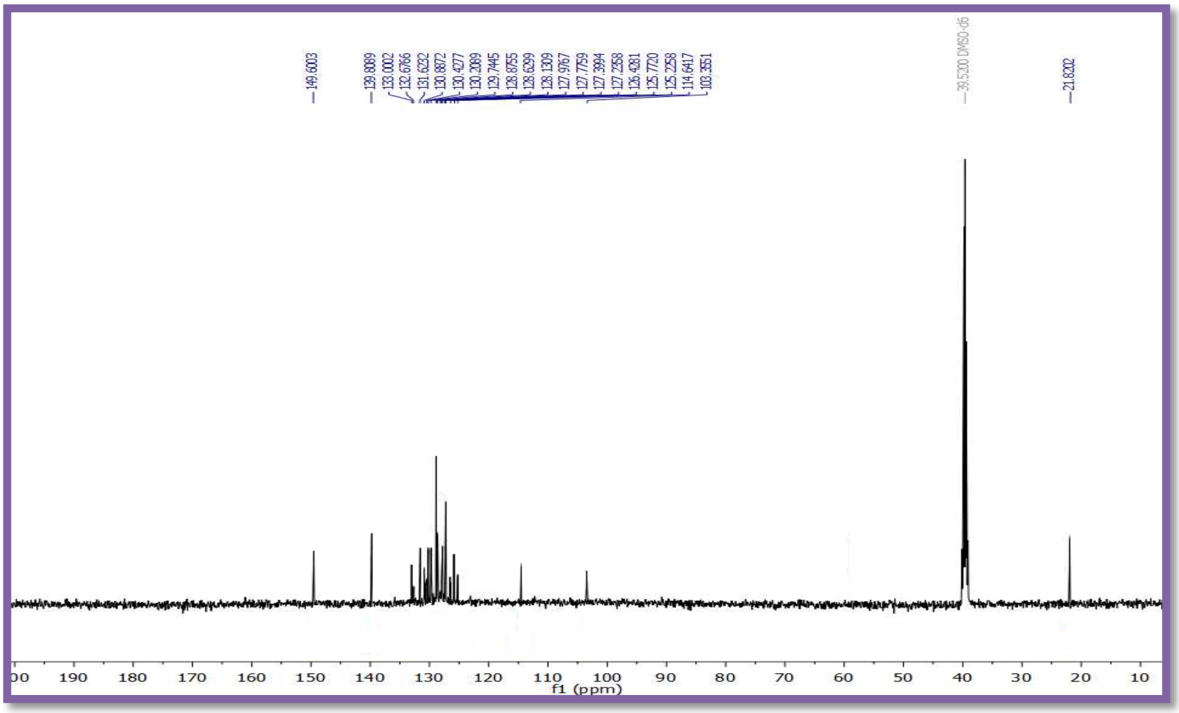

Figure S46. ^{13}C NMR spectrum of bis[(2-(3-(4-methylphenyl)-5-(2-chlorophenyl)-1H-pyrazol-1-yl)-3,5-dinitrophenyl)]tellurium dichloride (23).

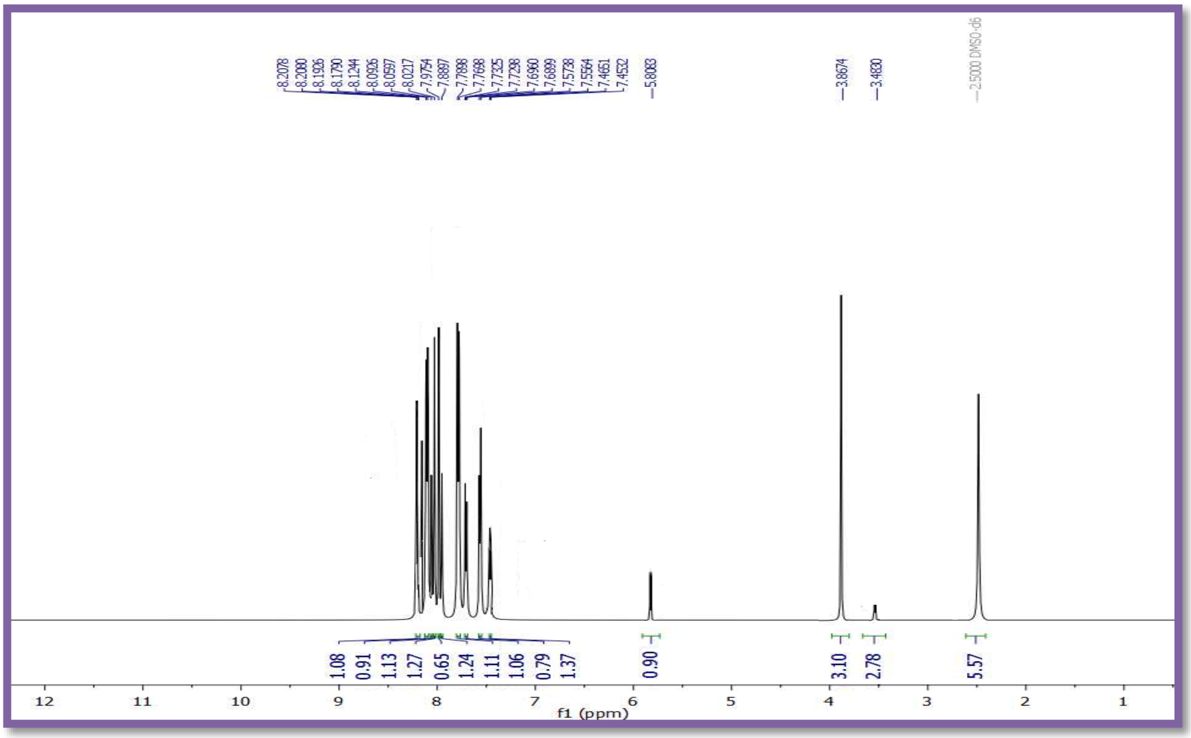

Figure S47. ^{1}H NMR spectrum of bis[(2-(3-(4-methoxyphenyl)-5-(2-chlorophenyl)-1H-pyrazol-1-yl)-3,5-dinitrophenyl)]tellurium dichloride (24).

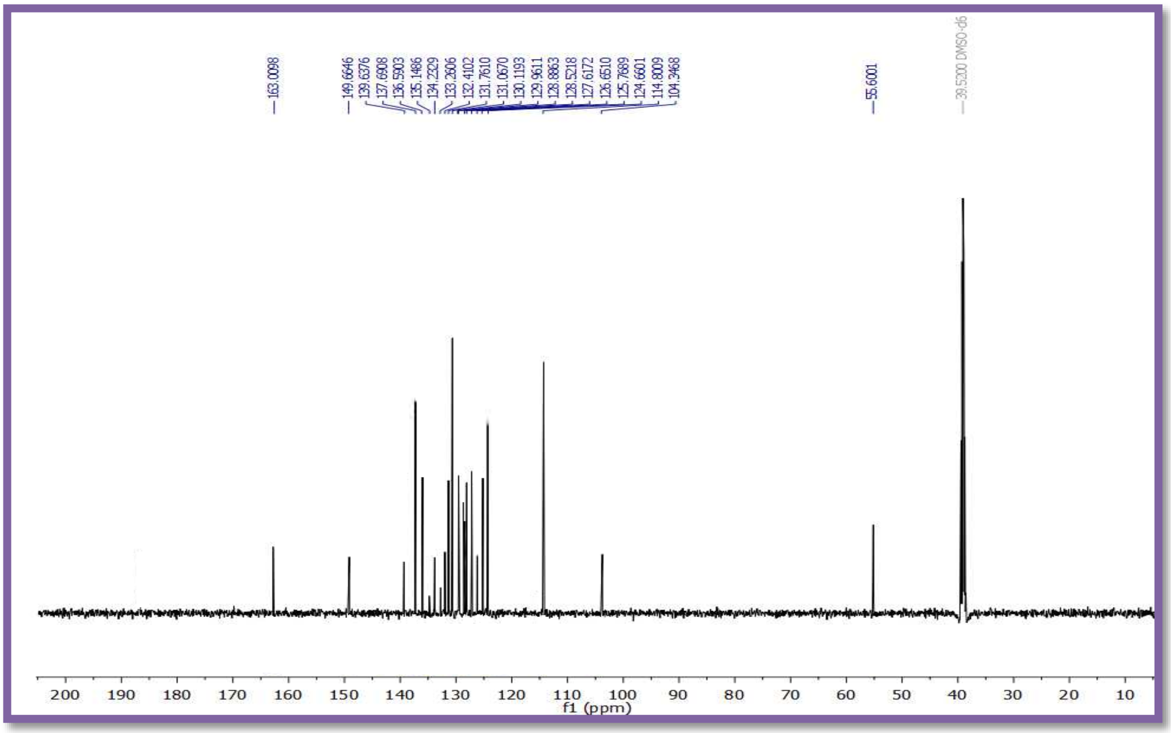

Figure S48. ^{13}C NMR spectrum of bis[(2-(3-(4-methoxyphenyl)-5-(2-chlorophenyl)-1H-pyrazol-1-yl)-3,5-dinitrophenyl)]tellurium dichloride (24).

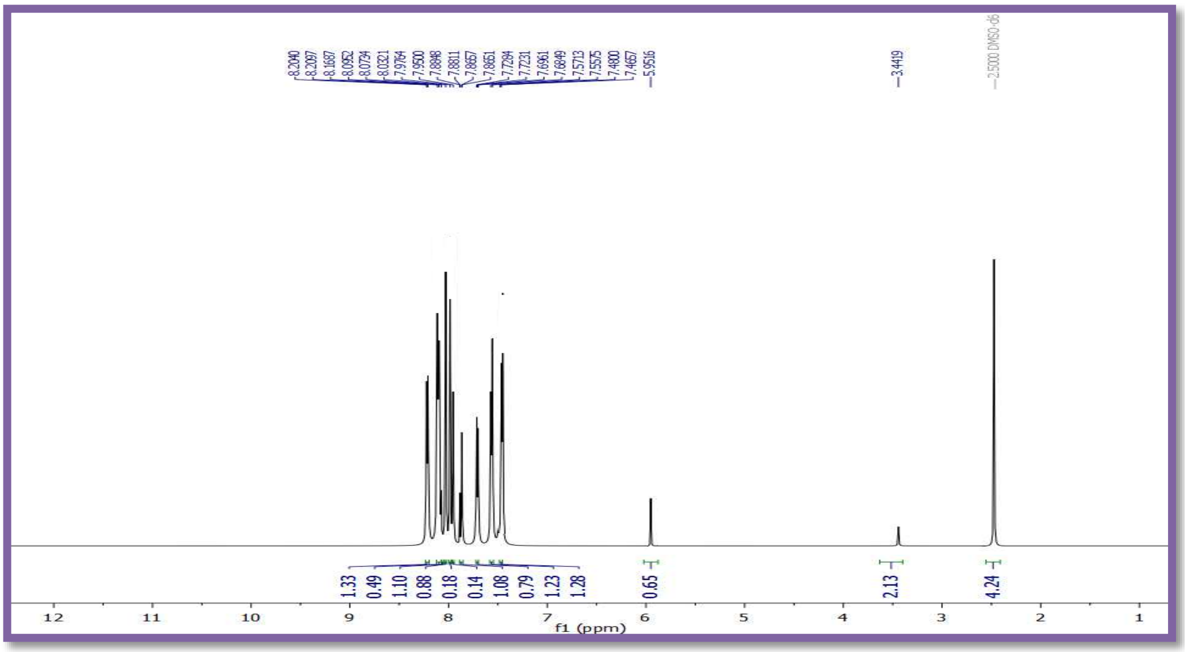

Figure S49. ^{1}H NMR spectrum of bis[(2-(3-(4-bromophenyl)-5-(2-chlorophenyl)-1H-pyrazol-1-yl)-3,5-dinitrophenyl)]tellurium diiodides (25).

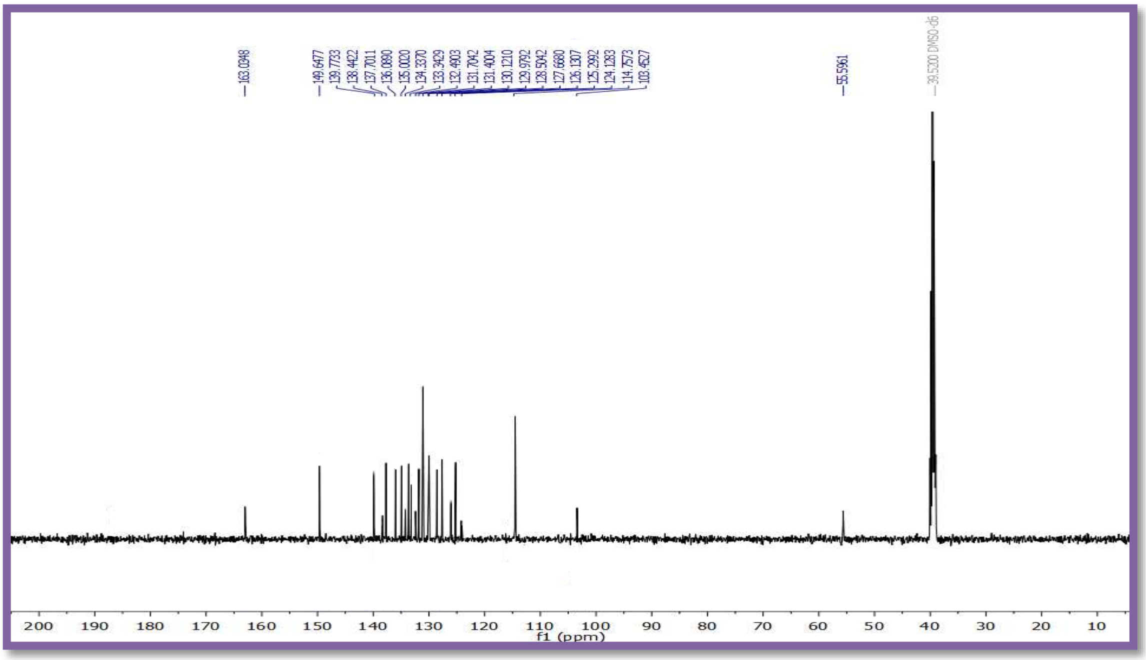

Figure S50. ^{13}C NMR spectrum of bis[(2-(3-(4-bromophenyl)-5-(2-chlorophenyl)-1H-pyrazol-1-yl)-3,5-dinitrophenyl)]tellurium diiodides (25).

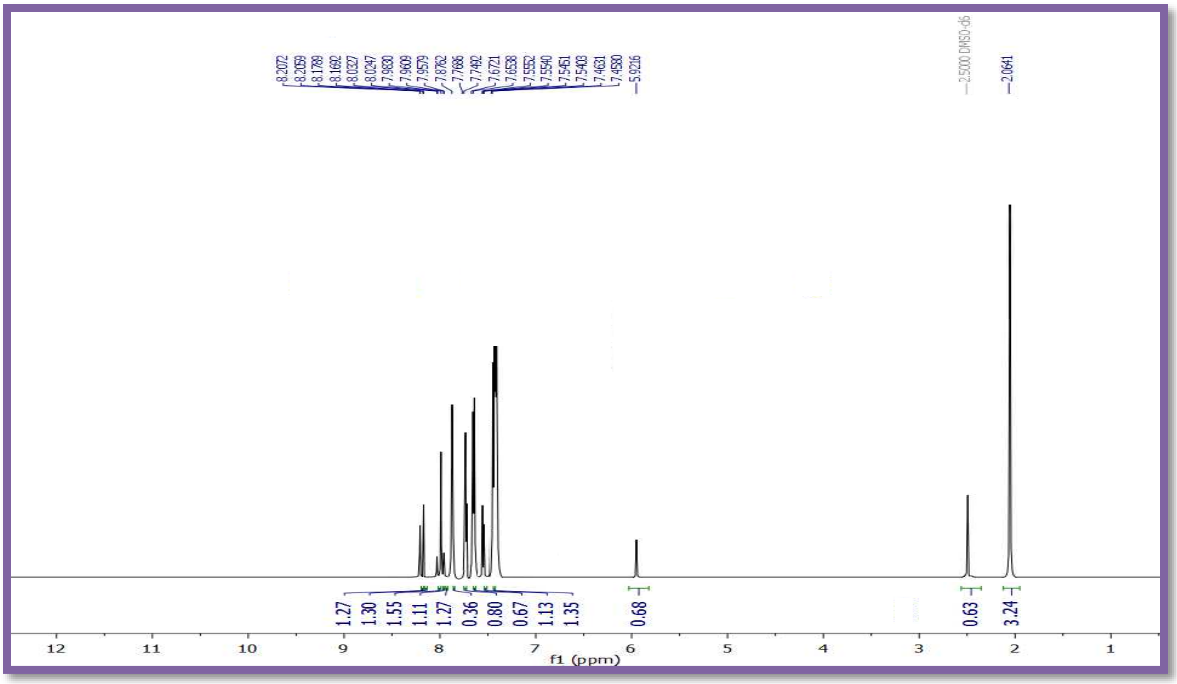

Figure S51. ^{1}H NMR spectrum of bis[(2-(3-(4-methylphenyl)-5-(2-chlorophenyl)-1H-pyrazol-1-yl)-3,5-dinitrophenyl)]tellurium diiodides (26).

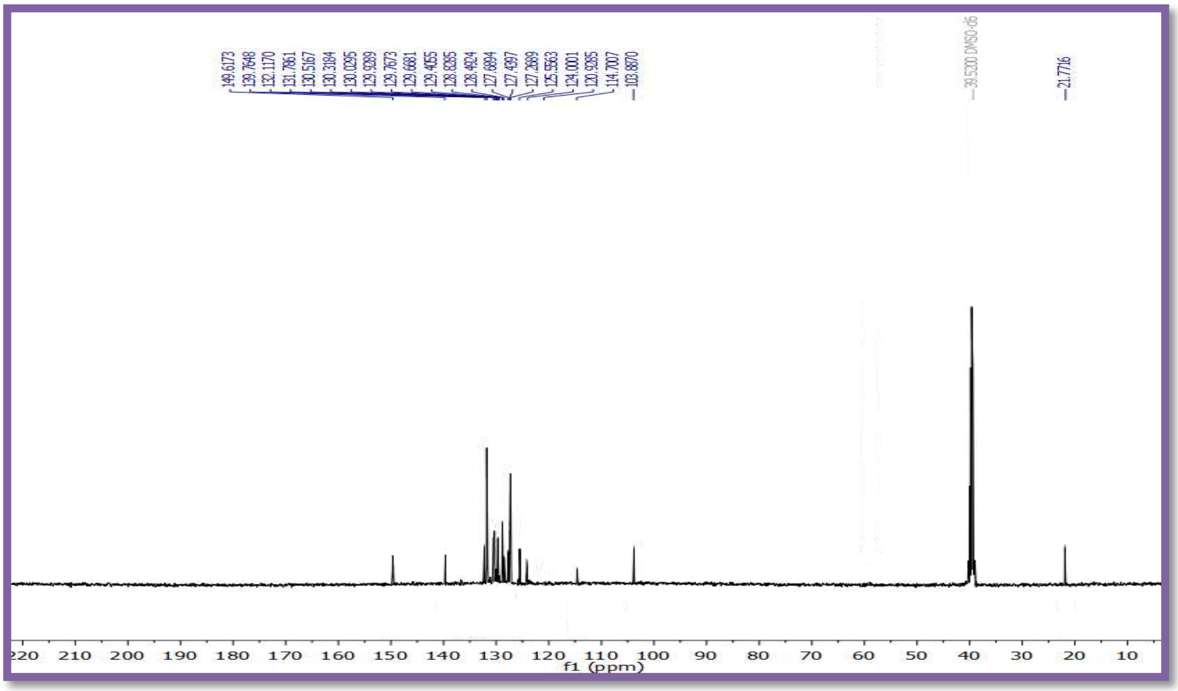

Figure S52. ^{13}C NMR spectrum of bis[(2-(3-(4-methylphenyl)-5-(2-chlorophenyl)-1H-pyrazol-1-yl)-3,5-dinitrophenyl)]tellurium diiodides (26).

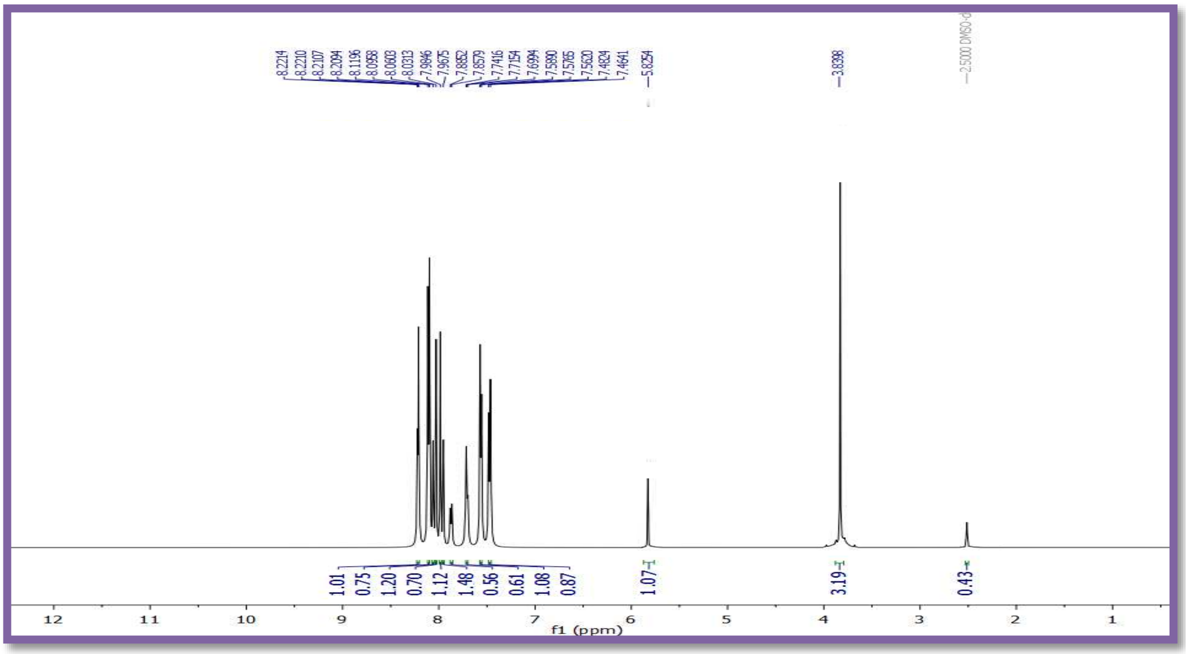

Figure S53. ^{1}H NMR spectrum of bis[(2-(3-(4-methoxyphenyl)-5-(2-chlorophenyl)-1H-pyrazol-1-yl)-3,5-dinitrophenyl)]tellurium diiodides (27).

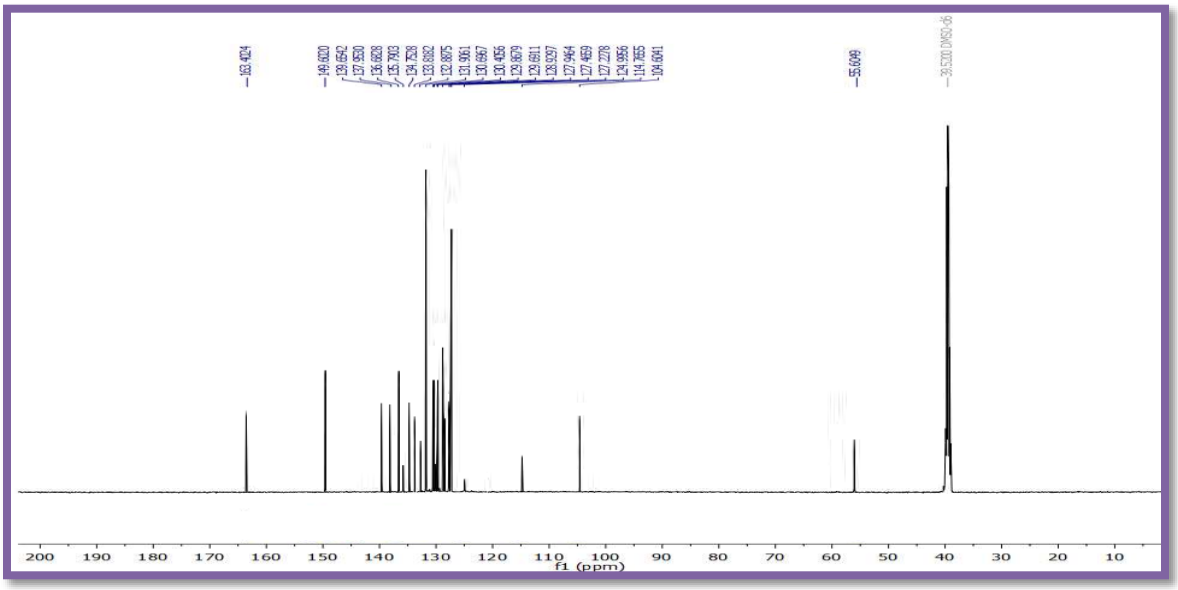

Figure S54. ^{13}C NMR spectrum of bis[(2-(3-(4-methoxyphenyl)-5-(2-chlorophenyl)-1H-pyrazol-1-yl)-3,5-dinitrophenyl)]tellurium diiodides (27).

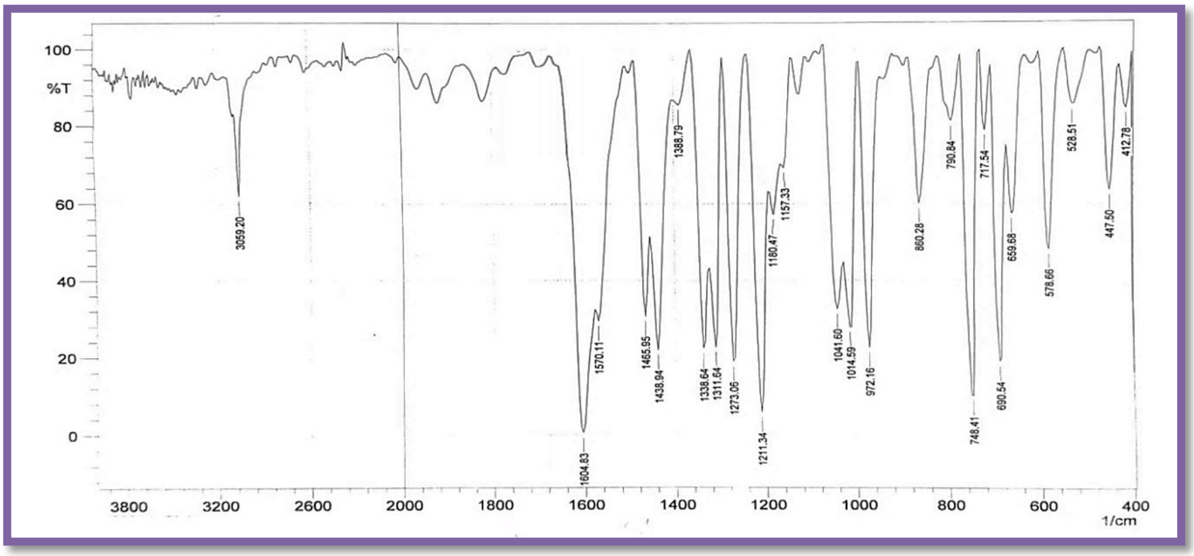

Figure S55. IR spectrum of 2-(3-(4-bromophenyl)-5-(2-chlorophenyl)-1H-pyrazol-1-yl)-3,5-dinitrophenyl) mercury (II) chloride (1).

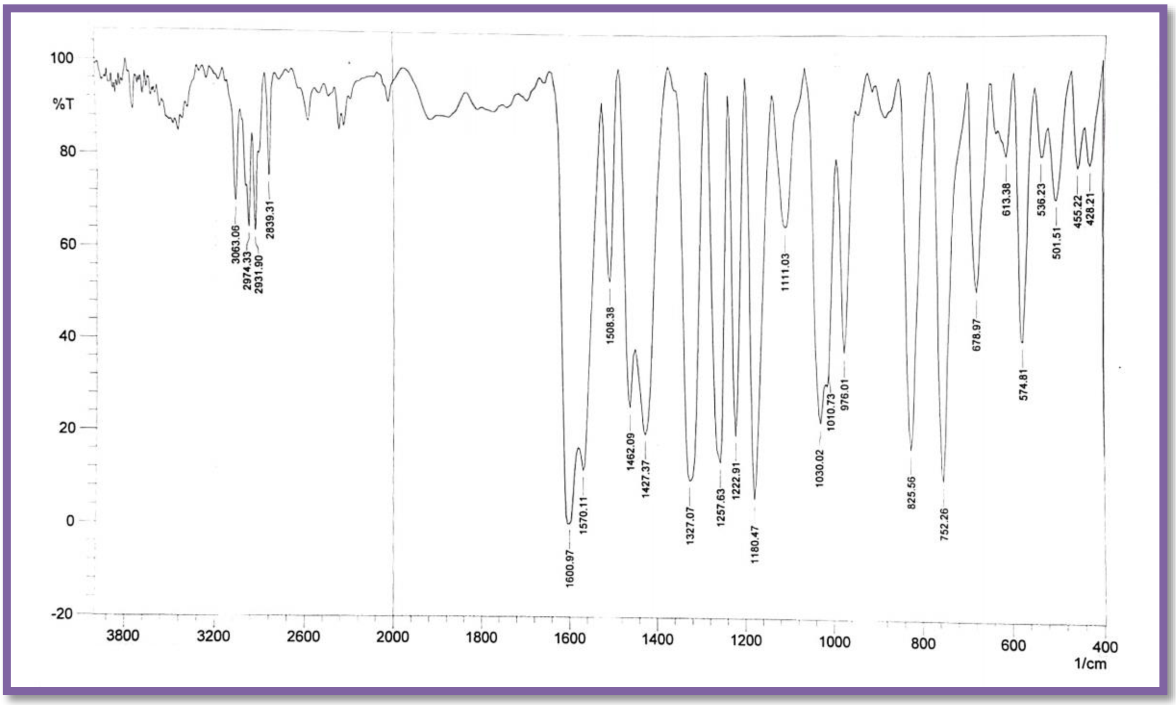

Figure S56. IR spectrum of 2-(5-(2-chlorophenyl)-3-(4-methoxyphenyl)-1H-pyrazol-1-yl)-3,5-dinitrophenyl)mercury(II) chloride (3).

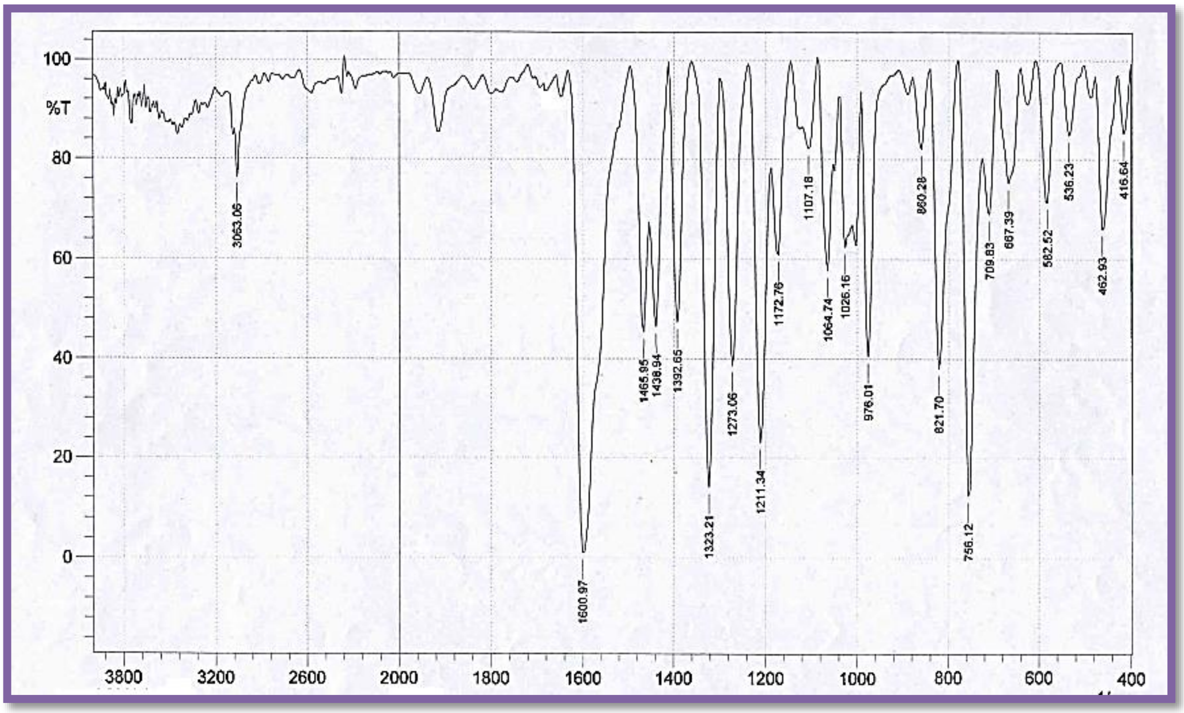

Figure S57. IR spectrum of (2-(3-(4-bromophenyl)-5-(2-chlorophenyl)-1H-pyrazol-1-yl)-3,5-dinitrophenyl)tellurium tribromide (4).

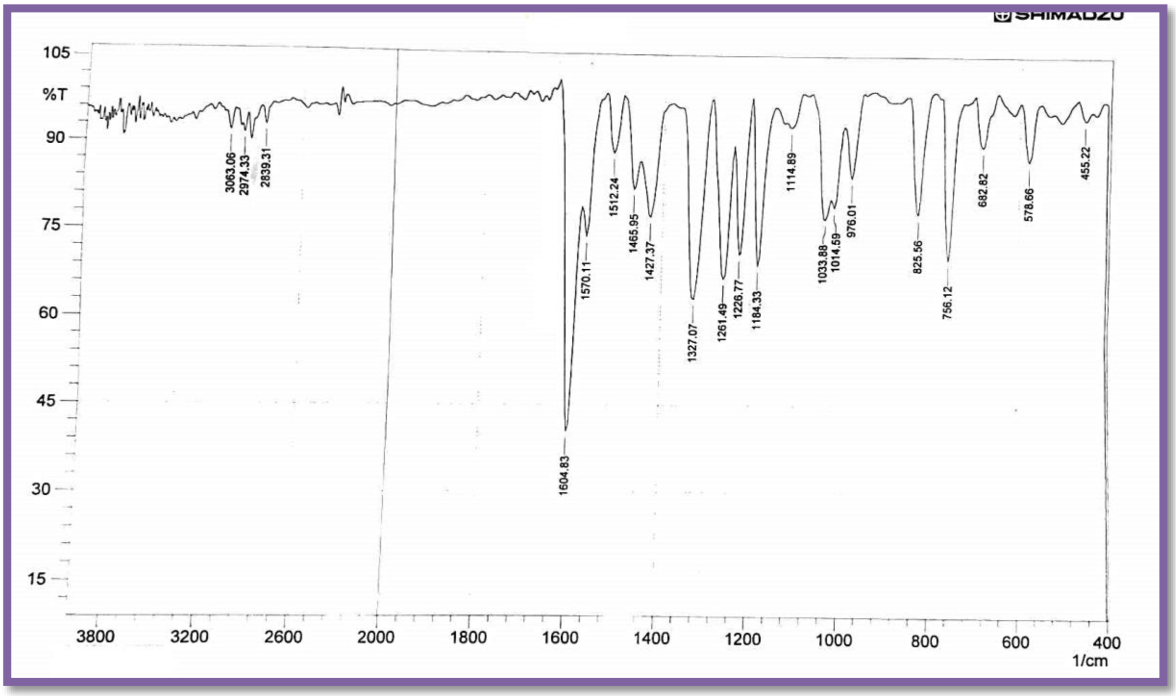

Figure S58. IR spectrum of (2-(5-(2-chlorophenyl)-3-(4-methoxyphenyl)-1H-pyrazol-1-yl)-3,5dinitrophenyl)tellurium tribromide (6).

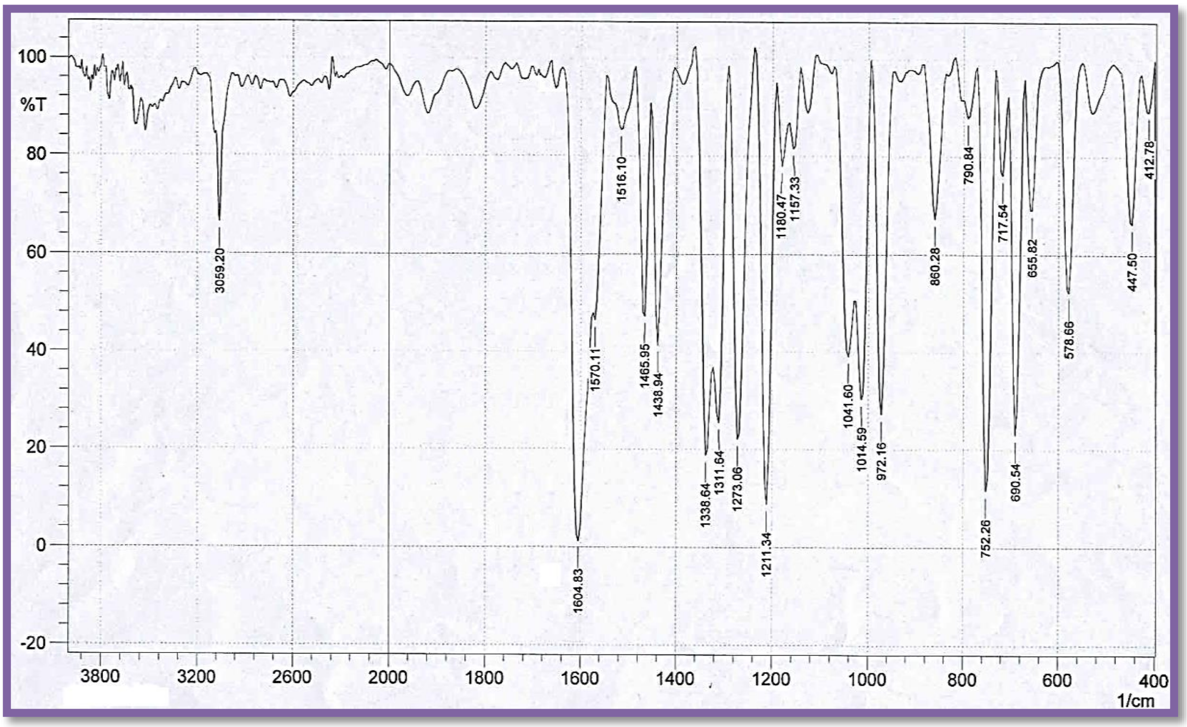

Figure S59. IR spectrum of bis[(2-(3-(4-bromophenyl)-5-(2-chlorophenyl)-1H-pyrazol-1-yl)-3,5-dinitrophenyl)]tellurium dibromide (7).

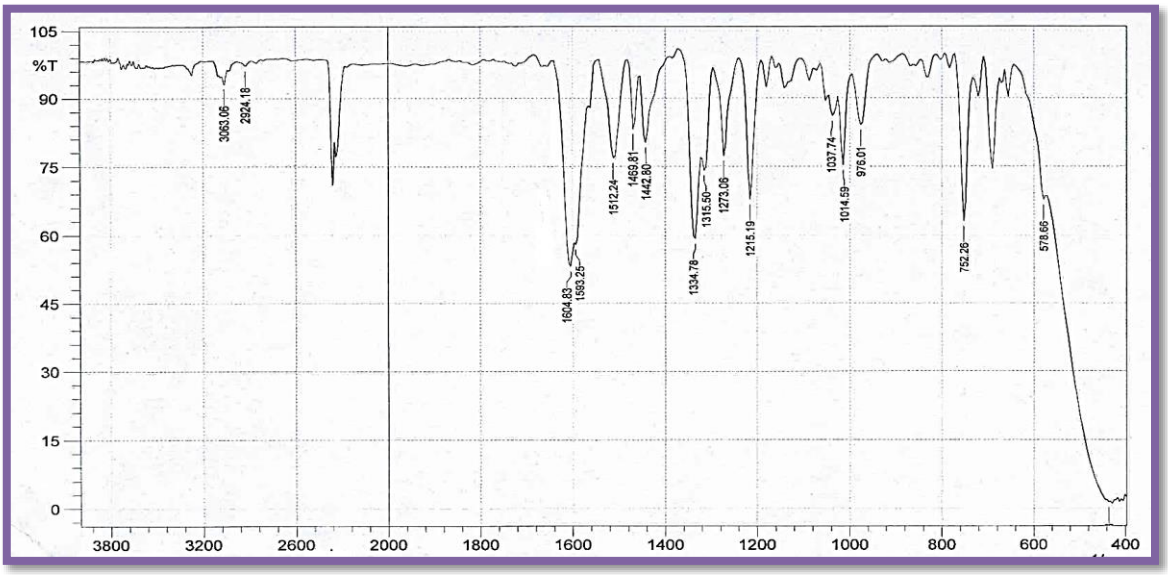

Figure S60. IR spectrum of bis[(2-(5-(2-chlorophenyl)-3-(4-methylphenyl)-1H-pyrazol-1-yl)-3,5-dinitrophenyl)]tellurium dibromide (8).

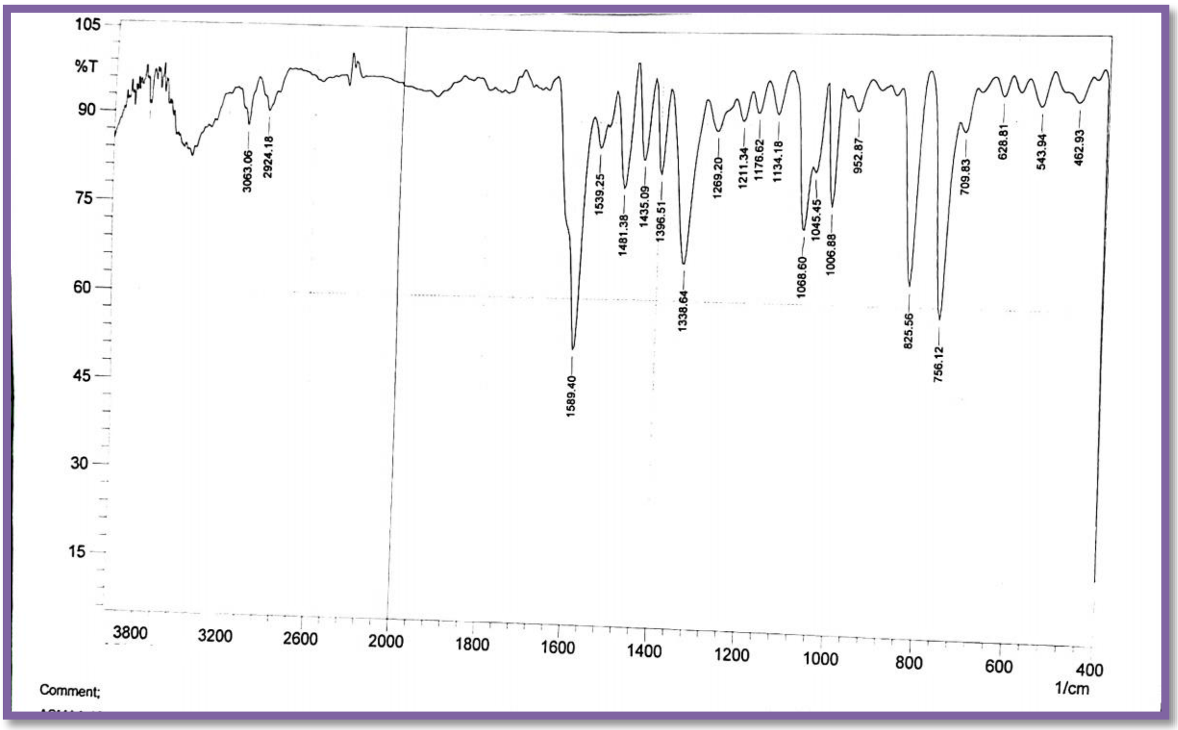

Figure S61. IR spectrum of bis[(2-(5-(2-chlorophenyl)-3-(4-methylphenyl)-1H-pyrazol-1-yl)-3,5-dinitrophenyl)] ditelluride (11).

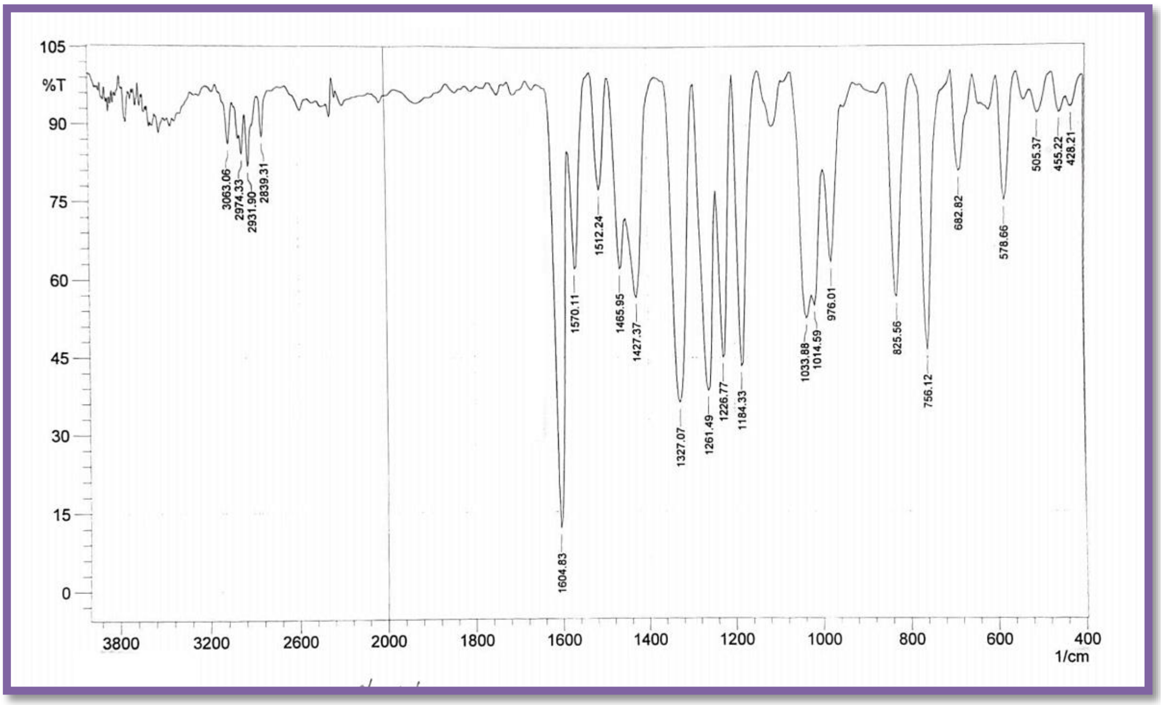

Figure S62. IR spectrum of **b**is[(2-(5-(2-chlorophenyl)-3-(4-methoxyphenyl)-1H-pyrazol-1-yl)-3,5dinitro phenyl)] ditelluride (12).

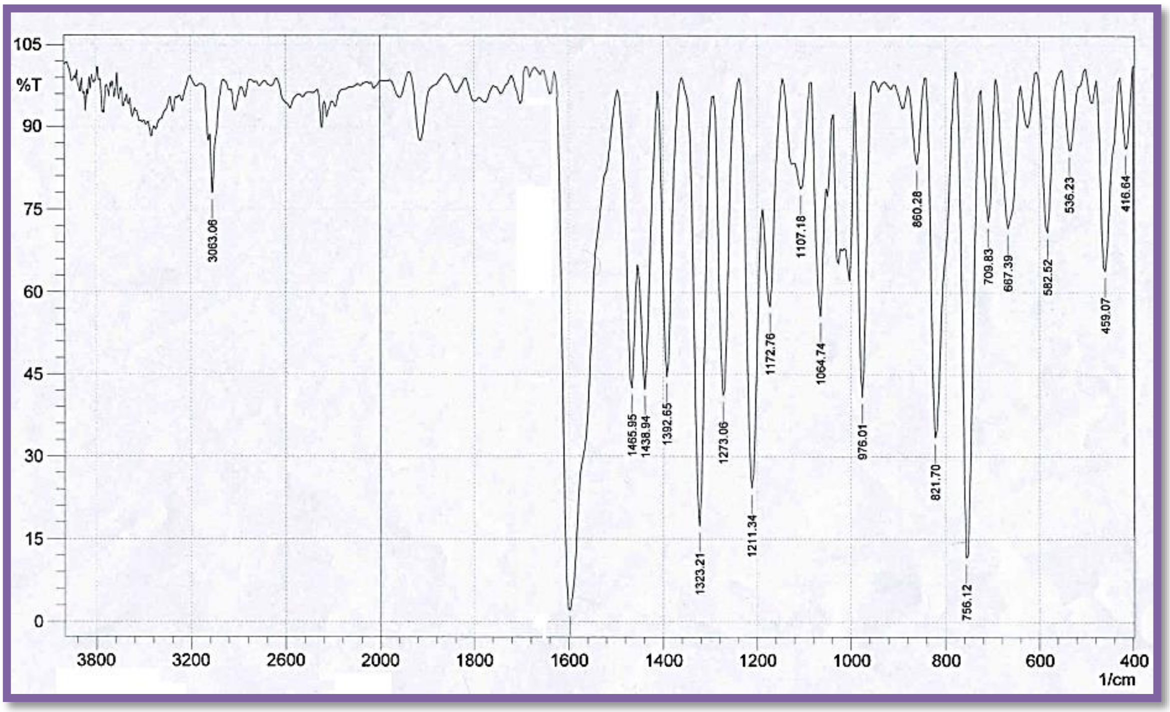

Figure S63. IR spectrum of bis[(2-(3-(4-bromophenyl)-5-(2-chlorophenyl)-1H-pyrazol-1-yl)-3,5-dinitrophenyl)] telluride (13).

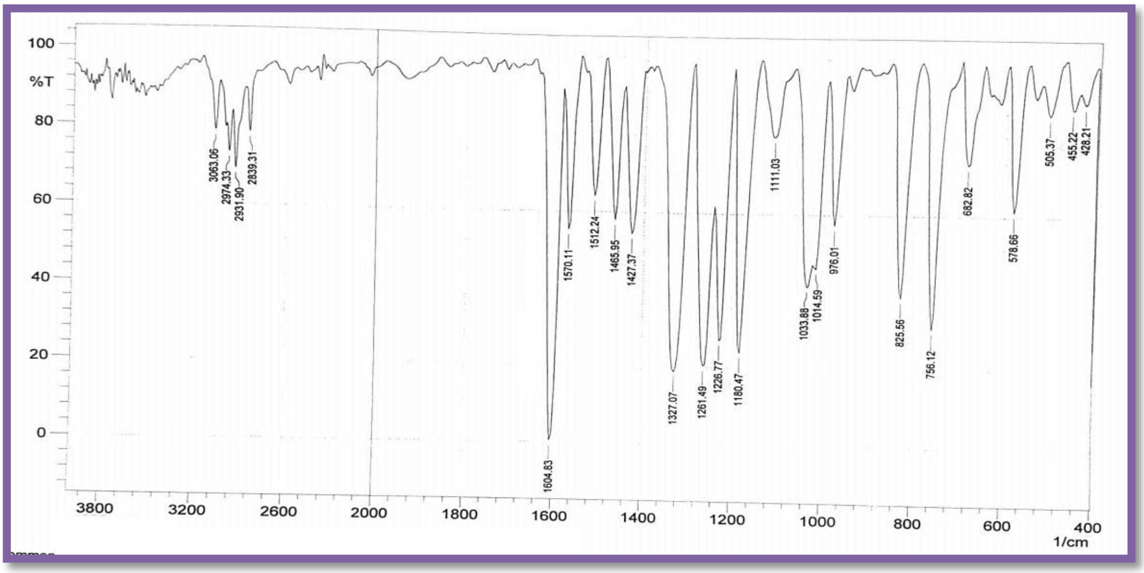

Figure S64. IR spectrum of bis[(2-(5-(2-chlorophenyl)-3-(4-methoxyphenyl)-1H-pyrazol-1-yl)-3,5-dinitrophenyl)] telluride (15).

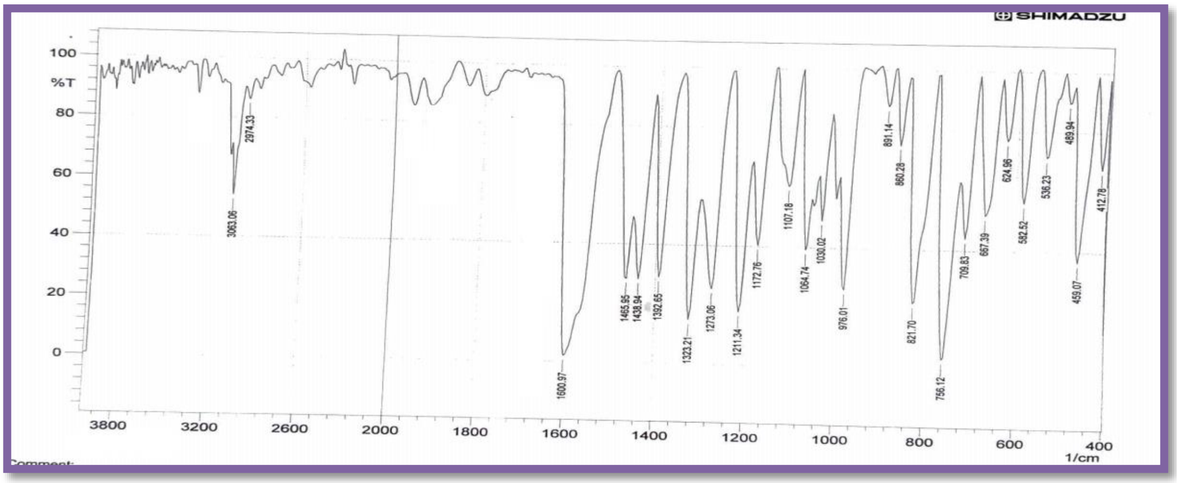

Figure S65. IR spectrum of (2-(3-(4-methylphenyl)-5-(2-chlorophenyl)-1H-pyrazol-1-yl)-3,5-dinitrophenyl)tellurium trichloride (17).

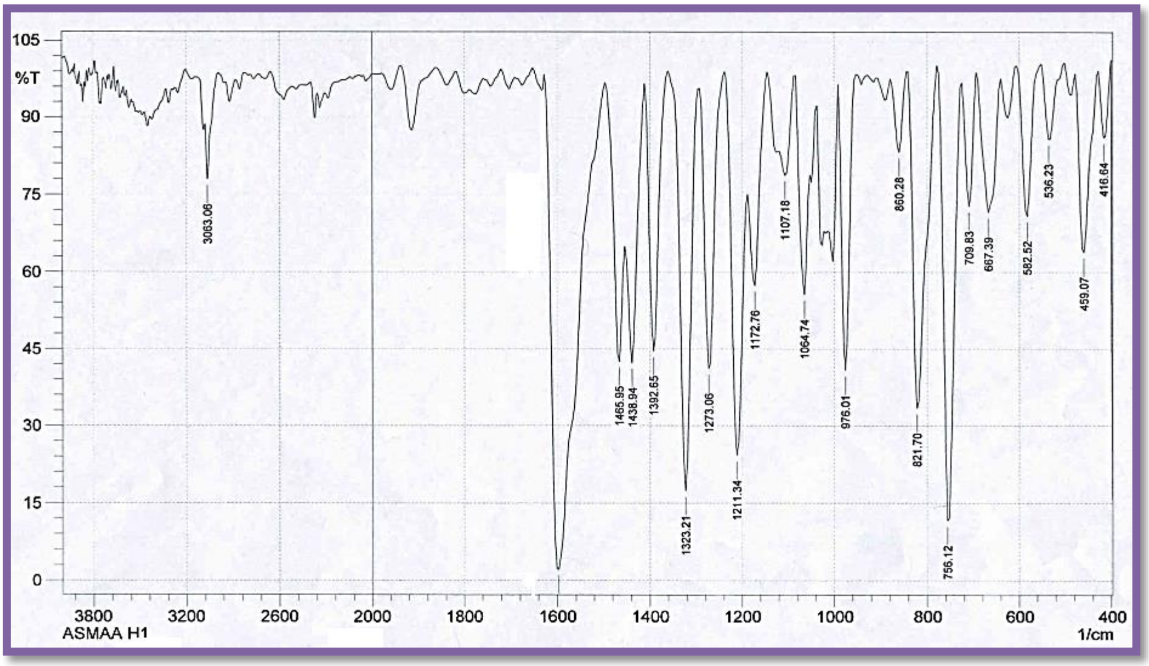

Figure S66. IR spectrum of bis[(2-(3-(4-bromophenyl)-5-(2-chlorophenyl)-1H-pyrazol-1-yl)-3,5-dinitrophenyl)]tellurium dichloride (22).

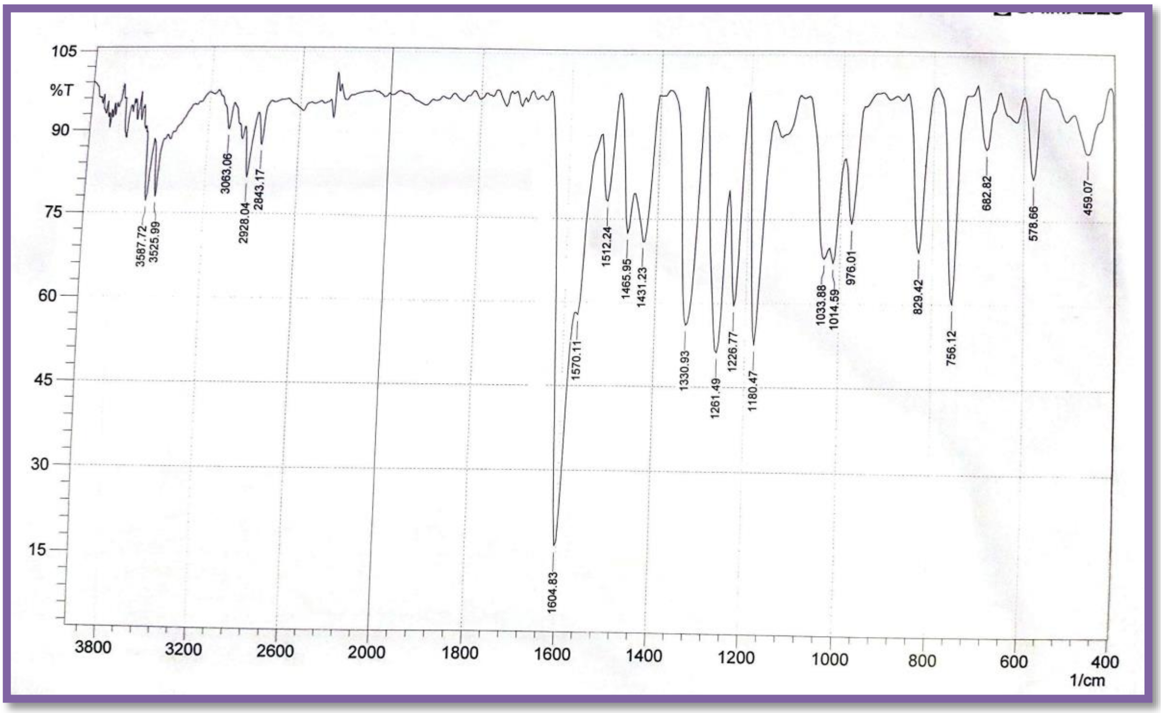

Figure S67. IR spectrum of bis[(2-(3-(4-methylphenyl)-5-(2-chlorophenyl)-1H-pyrazol-1-yl)-3,5-dinitrophenyl)]tellurium dichloride (23).

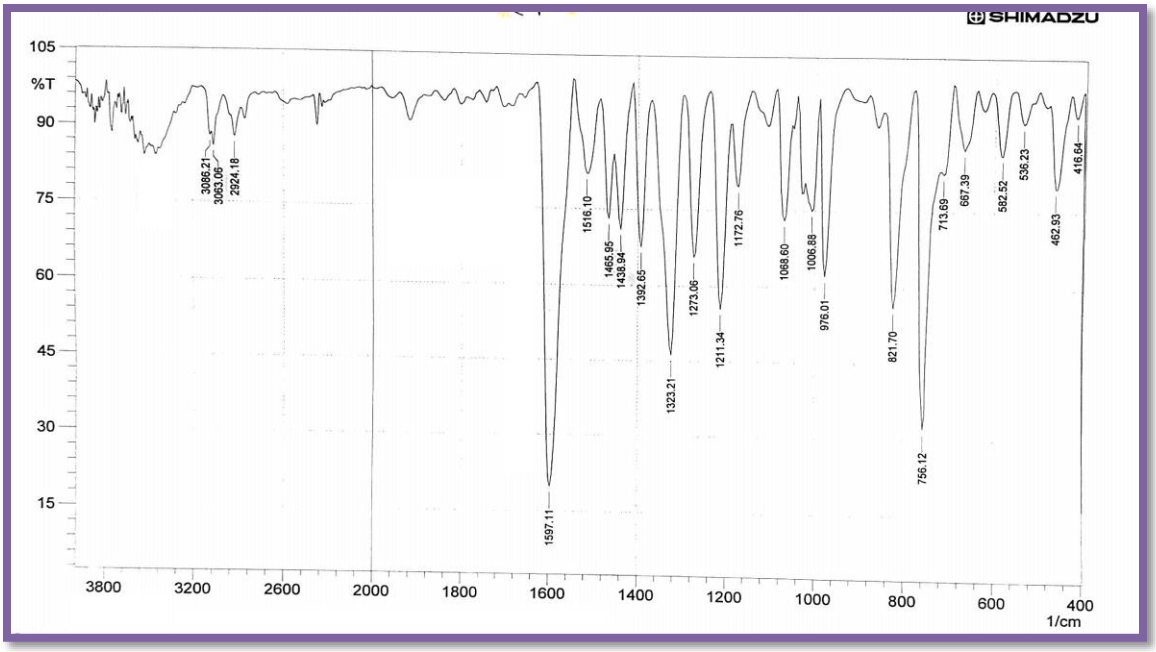

Figure S68. IR spectrum of (2-(3-(4-methylphenyl)-5-(2-chlorophenyl)-1*H*-pyrazol-1-yl)-3,5-dinitrophenyl)tellurium triiodide (20).

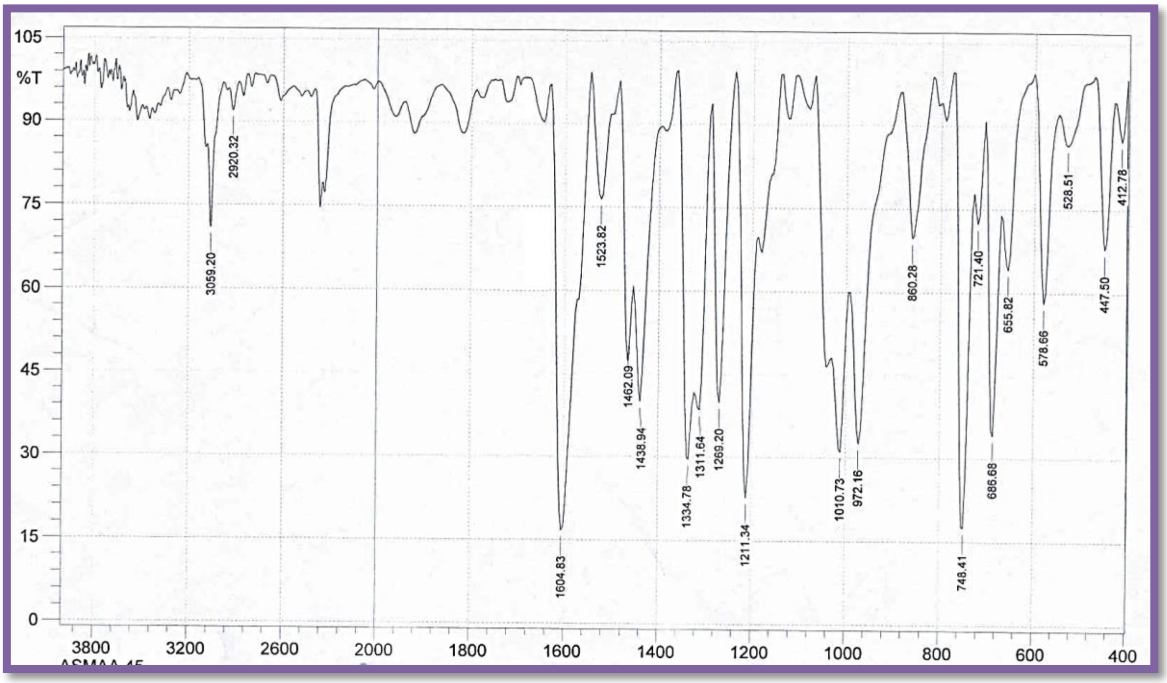

Figure S69. IR spectrum of bis[(2-(3-(4-methoxyphenyl)-5-(2-chlorophenyl)-1H-pyrazol-1-yl)-3,5-dinitrophenyl)]tellurium diiodides (27).
